# Hydroxyapatite coatings on cement paste as barriers against radiological contamination

**DOI:** 10.1038/s41598-023-37822-6

**Published:** 2023-07-10

**Authors:** Susan A. Cumberland, Andrea Hamilton, Joanna C. Renshaw, Kieran M. Tierney, Rebecca J. Lunn

**Affiliations:** 1 School of Geography, Geology and Environment, University of Leicester, Leicester, LE1 7RH UK; 2 Civil and Environmental Engineering, University of Strathclyde, Glasgow, G1 1XJ UK; 3 Scottish Universities Environmental Research Centre, University of Glasgow, Rankine Avenue, East Kilbride, G75 0QF UK

**Keywords:** Materials science, Nuclear energy, Civil engineering, Geochemistry

## Abstract

A novel method for precipitating hydroxyapatite (HAp) onto cement paste is investigated for protecting concrete infrastructure from radiological contamination. Legacy nuclear sites contain large volumes of contaminated concrete and are expensive and dangerous to decommission. One solution is to ‘design for decommissioning’ by confining contaminants to a thin layer. Current layering methods, including paints or films, offer poor durability over plant lifespans. Here, we present a mineral-HAp-coated cement, which innovatively serves as a barrier layer to radioactive contaminants (e.g. Sr, U). HAp is shown to directly mineralise onto a cement paste block in a layer several microns thick via a two-step process: first, applying a silica-based scaffold onto a cement paste block; and second, soaking the resulting block in a PO_4_-enriched Ringer’s solution. Strontium ingression was tested on coated and uncoated cement paste (~ 40 × 40 × 40mm cement, 450 mL, 1000 mg L^− 1^ Sr) for a period of 1-week. While both coated and uncoated samples reduced the solution concentration of Sr by half, Sr was held within the HAp layer of coated cement paste and was not observed within the cement matrix. In the uncoated samples, Sr had penetrated further into the block. Further studies aim to characterise HAp before and after exposure to a range of radioactive contaminants and to develop a method for mechanical layer separation.

## Introduction

The disposal of high and intermediate-level radioactive waste created during the decommissioning of existing nuclear infrastructure represents a global challenge. With nuclear power likely to form part of contemporary low-carbon transitions, actions that can be taken today to reduce future decommissioning waste from new-build plant, are vital. In particular, managing radioactive concrete represents a critical concern. Typical surface ablation methods employed to remove radioactive material from concrete include pressure washing^1^ and scabbling^2,3^; the latter liberating high levels of radioactive dust and posing health risks. The depth of the surface removed depends on the penetration of radionuclides which, if not remediated immediately after exposure, increases over time^4^. With the design lifespan of nuclear power plants being 30–40 years, and operational lifespans stretched longer, exposure timeframes are lengthy. Ingress arises as ordinary portland cement (OPC) can effectively scavenge radionuclides such as Cs, Eu and Sr, with observed penetrations of several mm in samples taken from buildings within nuclear plants^1,5–7^. Other typical radionuclides found within end-of-life nuclear infrastructure include 60-Co, 63-Ni, 90-Sr, 137-Cs, 129-I, U, Pu and Am, *inter alia*^5,8,9^.

Barriers applied historically to either dry or wet concrete surfaces to reduce or prevent deeper penetration of radionuclides vary in their effectiveness. For example, samples taken from 60-year-old Hunterston-A infrastructure, showed that while Sr was associated with the historic titanium-based paint layers used to seal wet concrete surfaces, radioactive Sr was also present in the concrete beneath the Ti paint layer, indicating that breaching had occurred^7^.

Epoxy coated cement has been used to protect and repair concrete but may degrade over time and surfaces may require treatment to fully decontaminate^10–12^. Strippable layers e.g. latex or complex polymer based compounds, applied post contamination aim to remove surface bound radionuclides, rather than prevent contamination^13,14^. ALARA 1146 was the first commercially available strippable layer described as a hydrophilic vinyl butyl solvent-free coating^15,16^, and since 1999 more products are now commercially available (e.g.^14,16–18^). Recently reviewed by Wang et al.^17^, the benefits of strippable layers are their applications to multiple situations, particularly for non-porous surfaces such as steel and aluminium^13,19^. However, there are few reports for porous surfaces such as concrete and pavements and where applied, they were found difficult to remove^15,16,19,20^.

While paint or epoxy layers can reduce the volume of concrete waste, disadvantages arise in their long-term mechanical durability, potential for removal and suitability for disposal (i.e. its stability) as radioactive waste^8,21^. The problem of treating contaminated surfaces is particularly notable in areas of high radiation with restricted access for maintenance, such as fluid-filled pipes and waste storage vaults, leading to substantial mechanical and chemical degradation^3^. Therefore, decontaminating inaccessible areas requires a more practical approach.

In this paper we propose the development of a hydroxyapatite (HAp, [Ca_10_(PO_4_)_6_(OH)_2_]) surface coating as a novel method for inhibiting the ingress of radionuclides into concrete nuclear infrastructure. Phosphate-based minerals have the potential to be mechanically durable and have the capability to fix and stabilise radionuclides through sorption and mineral replacement^22,23^. HAp, in particular, can take up significant amounts of Sr and U(VI)^6,23–29^.

To create a durable HAp-based concrete coating, HAp crystals must nucleate and grow on the surface of hardened cement pastes. The synthesis of HAp as layers has been extensively investigated for medical applications due to its compatibility with bone and teeth^22,30^. Medical research has shown that for HAp synthesis, nucleation in solution at low temperature is rapid and easy to achieve, whereas mineralisation onto surfaces is challenging^31–33^. To synthesise HAp layers on a surface, certain criteria need to be met^34^. Negatively charged surfaces are considered favourable for HAp formation^35,36^; For example, in bodily fluids, with a pH of 7.35–7.45, and salt strength about 100 mM^37^, HAp will form on titanium bone implants but not on positively charged aluminium implants^35,38^. Elsewhere in the literature, HAp has been attached to surfaces such as marble^39^, but most promising are silica-rich surfaces^32,33,38^.

Surface charge is not the only consideration for successful HAp precipitation; despite their silica-rich surfaces, quartz and soda glass make poor scaffolds^32,38^. Medical researchers have shown that both surface charge *and* Si type, particularly hydrated silica (silanol, Si–OH) are important for HAp nucleation^35–37^. These findings have led to the provision of alternative surface scaffold materials. Silica-based sol-gels^38,40^ and modified glasses, such as bio-glass, which is enriched in P and Ca (Na_2_O–CaO–SiO_2_–P_2_O_5_), are considered to be suitable for bone growth in the laboratory^32,33,38^. The release of sodium from the bio-glass modifies the surfaces providing Si–O^−^… H^+^ whilst increasing the interfacial pH resulting in a hydrated silica-gel layer capable of trapping Ca ions in its porous structure^37^.

In the following research we draw on the medical literature to develop, and test the efficacy of, a novel method for HAp-coating of hardened cement-based structures. We investigate two silica-based scaffolds applied to hardened cement block surfaces. The coated blocks are soaked using two different strategies in (phosphate-amended) salt solutions. We demonstrate that HAp-coating of the hardened cement surface is successful at preventing strontium ingress into the cement paste.

### Investigation of HAp formation from different solution strengths and soaking times

**Figure 1:**
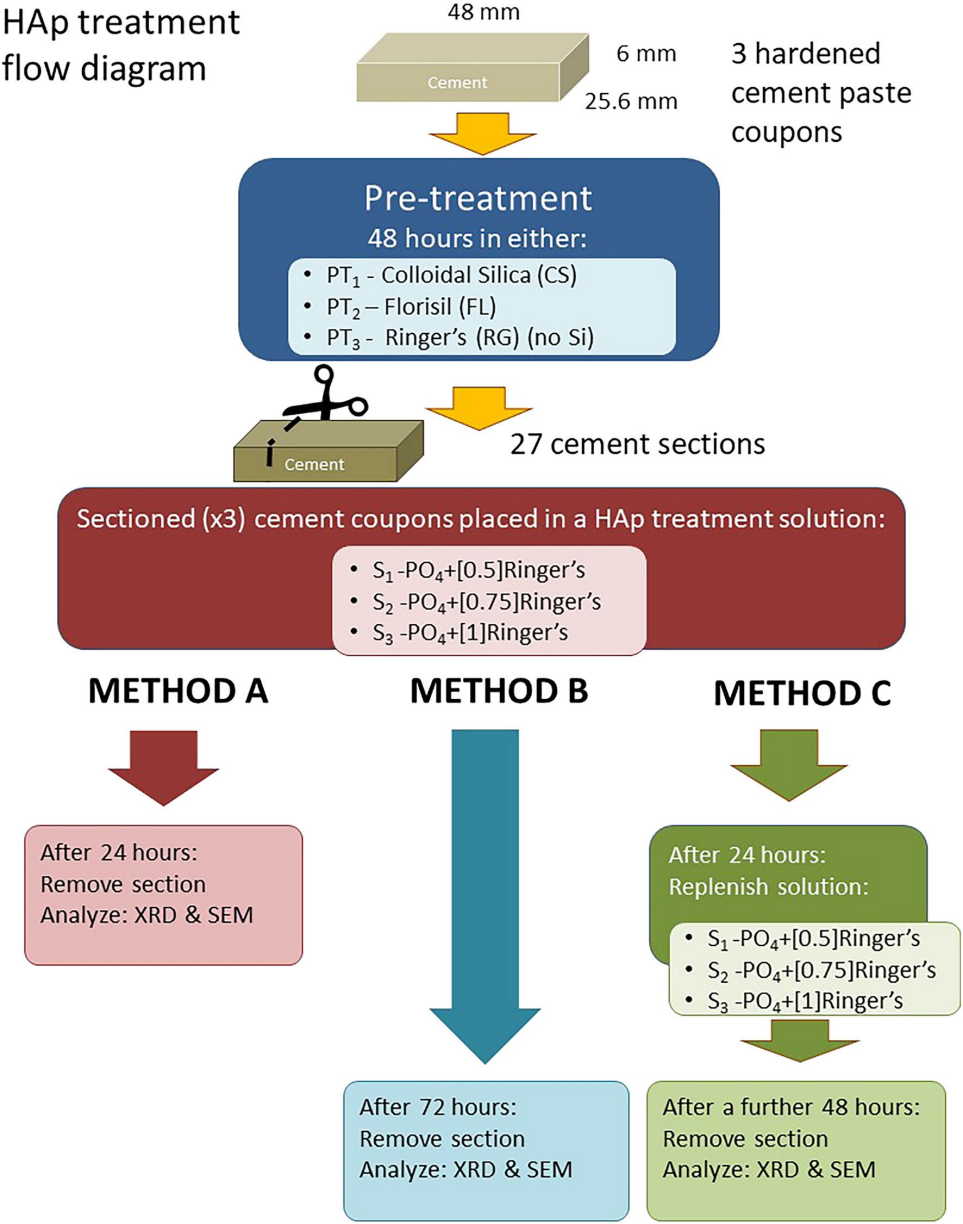
Schematic drawing of HAp formation experimental process and treatment of hardened cement paste coupons.

Details of the experimental methodology are provided in the methods section and are summarised in Fig. 1 and TableS1. We took three-week aged hardened cement paste blocks and applied the scaffold by a two-day pre-treatment with either a silica-based solution (PT1, PT2) or a control solution containing no silica (PT3). These blocks were then removed, sub-sectioned and placed in a phosphate buffered Ringer’s solution (PO_4_-Ringer’s) at different Ringer’s concentrations (S_1_, S_2_ or S_3_), where for each solution the P concentration was kept constant and the Ca concentration increased (i.e., 0.044, 0.065 or 0.088 g L^− 1^ Ca). For each solution (S_1_, S_2_ or S_3_), various soaking methods of the blocks were investigated to promote HAp precipitation: Method-A, one-day soaking; Method-B, three-day soaking; Method-C, soaking for 24 h followed by soaking in a fresh PO_4_-Ringer’s solution for a further 48 h. The hardened cement paste blocks were then examined for HAp formation by X-ray diffraction (XRD) and scanning electron microscopy (SEM) with energy dispersive spectroscopy (EDS). The most suitable method from the above was subsequently identified and used to prepare larger HAP-coated cement paste blocks for testing of Sr ingression, by soaking them in a solution of 1000 mg L^− 1^ Sr.

## Results

### Characterisation of HAP layers by XRD and SEM

**Figure 2:**
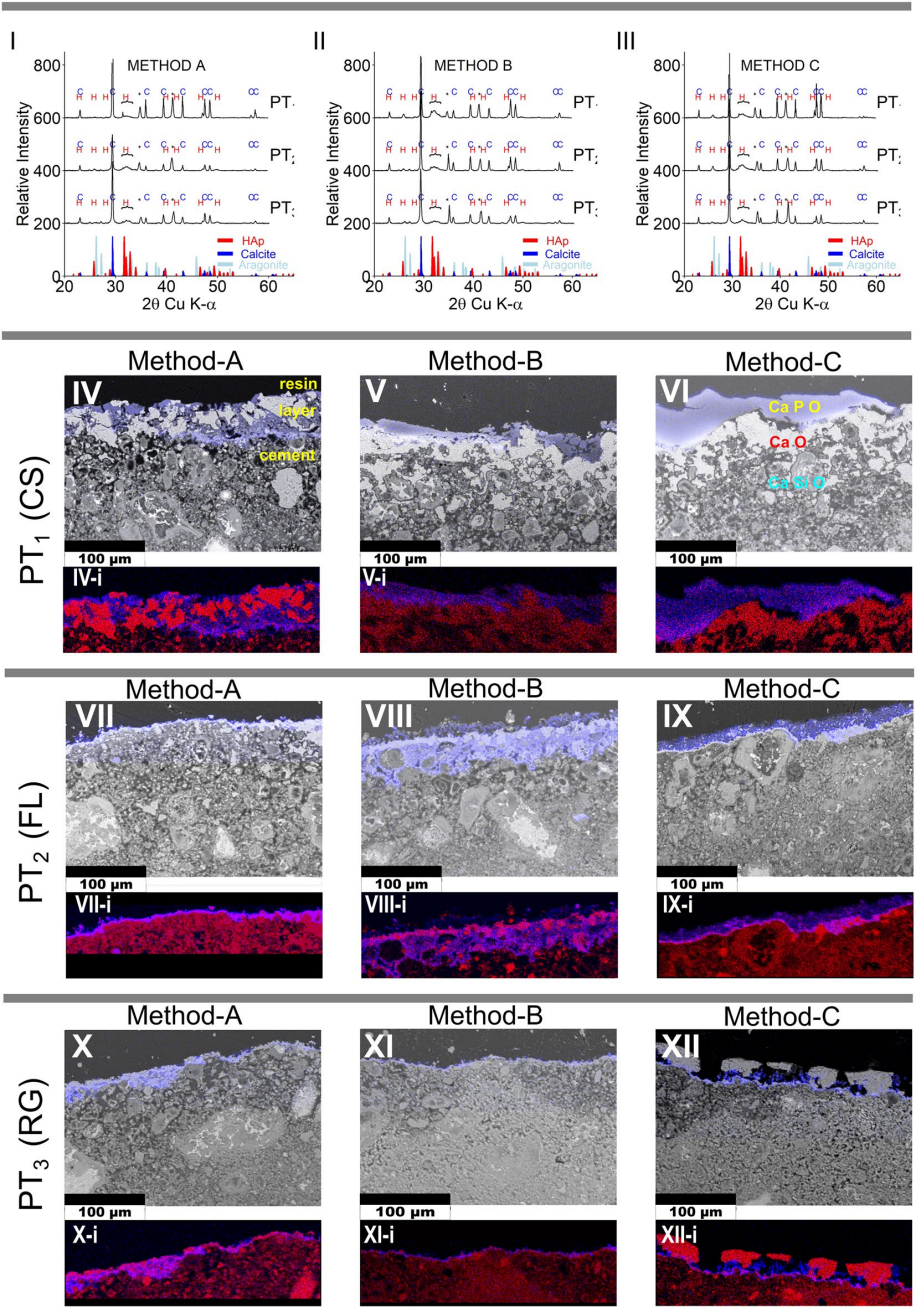
XRD patterns and cross-sectional SEM–EDS images of the HAp coated hardened cement pastes according to experimental method in grid format. Top (**I, II, III**); are XRD patterns taken from the solid face of the hardened cement paste surfaces and correspond column-wise for each method A, B or C. C and H above the XRD patterns indicate the calcite or HAp peaks respectively. Additional diffraction peaks observed at 34.6–35.3 and 41.1–41.7 2Θ Cu K-α, marked with an asterisk are likely an artefact from the Al sample holder and is therefore not considered as part of the sample. SEM–EDS images; column-wise, left to right represent soaking methods A, B and C, and row-wise; Silica and control pre-treatments; PT_1_ = Colloidal silica, PT_2_ = Florisil, and PT_3_ = Ringer’s. Where the blue and grey composite SEM–EDS images (**IV to XII**) show the distribution of P (blue) overlain on the backscatter background image (grey). Images (**IV-i to XII-i**) are SEM–EDS element map overlays of Ca(red) and P(blue) of the layer above (**IV to XII**). Maps and images were created using Oxford Inca and processed using ImageJ software. All images are 320 × 231 μm (w × h). Scale bars for images are 100 μm. Top right SEM image (**VI**), indicates elements present in the layers, carbon is present throughout.

XRD results indicate the calcium phosphate phase formed was hydroxyapatite (Fig. 2 I, II and III, Fig. S-1, Fig. S-2) which is the most stable calcium phosphate phase to form in aqueous solution at pH > 4^39^, Metastable phases such as octa calcium phosphate (OCP, [Ca_8_H_2_(PO4)_6_ּ.5H_2_O]), can form and the similarity of the XRD patterns for HAp and OCP is known to make conclusive identification challenging^39^. In this case, the pH of the solution used to precipitate the first layer was 9.3–11.1 after 24 h (varying with surface pre-treatment) and HAp preferentially forms over OCP at pH > 9.25^41^. During the second phosphate treatment as part of method C, the solution pH is lower (7.2–9.6) OCP is more likely to form but could easily convert to HAp. OCP has two characteristic reflections (above 5 degrees 2θ Cu K-α) at 9.45 and 9.8 degrees 2θ Cu K-α that are not present in HAp and these reflections are visible in only one sample (PT2, S2, METHOD C). TOPAS (v5.0, Bruker) was used to quantify the data and the quality of the fit for HAp (R-Bragg and refined mineral density) was better than for OCP. In addition to HAp, calcite (CaCO_3_) and aragonite (CaCO_3_) are also present. We do not claim the HAp formed is stoichiometric as it could be carbonated in this system and forms in the presence of other ions (e.g. K, Na, Cl) in the Ringers’ solution. Semi-quantitative results from Rietveld refinement show that PT1 and PT2 produced more HAp (median 55 and 54 wt. % respectively) than PT3 (median 37 wt. %) (Table S2). Method C produced more HAp (median 54 wt. %) compared to methods A and B (median 47 wt. % and 46 wt. % respectively). Hardened cement paste samples viewed in cross-section under SEM show the substrate and precipitated layer from the backscatter image overlaid with a blue (false colour) EDS element map for phosphorous to indicate where HAp formed (Fig. 2 IV to XII). Using SEM-energy dispersive X-ray spectroscopy (SEM–EDS) Ca maps in red are overlaid with P maps in blue, showing the presence of HAp, calcite and portlandite though portlandite was not present in the XRD analyses, probably due to limited depth penetration of laboratory XRD (Fig. 2 I to III, Fig.S-1, Fig. S-2).

SEM backscatter images and EDS element P maps of the coated hardened cement pastes in cross-section were used to assess the thickness of the HAp. HAp was more dominant on rough surfaces than on smooth surfaces across all pre-treatments (CS, FL and RG). Extremely smooth surfaces, e.g., where the surface was in contact with the mould used to form the cement samples, however, showed little evidence of any HAp present. Because of the lack of HAp on smooth surfaces, manual surface roughening was adopted for experiments involving the larger cement paste cubes. HAp layers were thicker on surfaces which had been pre-treated with silica-rich suspensions PT1 and PT2, (Fig. 2) compared to no silica treatment (PT3). HAp layers in samples from METHODS B and C (after 72 h) are thicker compared to METHOD A (24 h). Some layers appear to be more integrated with the underlying hardened cement paste matrix than others. Both the CS and Florisil produced uniform coatings, with the CS having the most discrete surface contact between the coating and the hardened cement paste, particularly using METHOD C, which appears to be a purely surface coating.

### HAp thicknesses using SEM–EDS P-element maps

For each treatment, the thickness of the HAp layers was determined from multiple measurements of the width of the phosphorous (P) band in the SEM–EDS (Oxford, Inca) P element maps, for a single sample image (Fig. 2; Table S3). HAp thickness at the hardened cement paste surfaces ranged between 0.3 and 82.7 µm (Table S4) (n > 40 measurements per map, at approximately 8–10 pixel intervals). Because data are not normally distributed (*Shapiro, R*), non-parametric medians and median absolute deviation (MAD) are therefore reported, where the MAD is equivalent to the interquartile range.

### Effect of treatment type on HAp thickness

**Figure 3:**
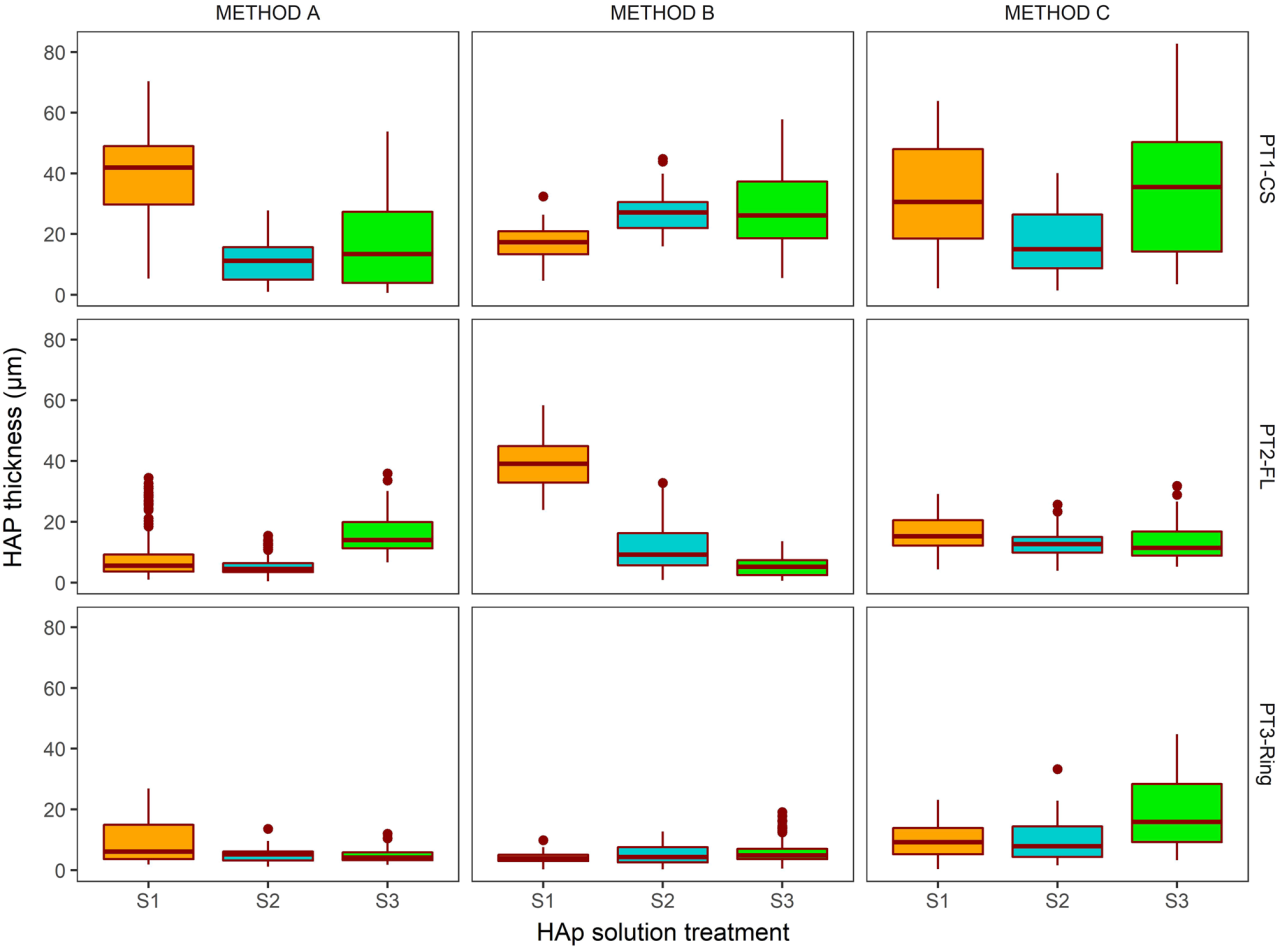
Boxplots showing the distribution of HAp coverage measured on the surface of the treated hardened cement pastes. The bar represents the median, the tails represent the spread of the data and the dots are outliers (i.e. 1st or 3rd quartile ± 1.5 × IQR). S1 = solution 1, S2 = solution 2, S3 = solution 3.

Boxplots showing the median value and the variability for each sample thickness are presented in Fig. 3. The median values of the data which are ranked in order of greatest thickness can be found in the supplementary data (Table S3). Hardened cement paste blocks that received the silica-based pre-treatments (PT_1_ and PT_2_) had thicker HAp layers than those that had not (13.6 ± 12.8 µm MAD compared to 5.64 ± 3.7 µm MAD). While the PT_2_ (FL) showed promise as a silica pre-treatment, the technique using colloidal silica showed more consistency in HAp thickness. There was no advantage found in increasing the Ca (Ringer’s) concentration in HAp solution treatment (S_1_, S_2_ or S_3_).

For all pre-treatments and ringer’s concentrations used, thicker HAp layers were generally observed through longer soaking times: 72 h for METHODS-B and C compared to 24 h for METHOD-A. There was some advantage seen in replenishing the solutions after 24 h (METHOD-C) compared to continuing the treatment in the same solution (METHOD-B). HAp thickness from METHOD-C was 13.89 ± 8.87 µm MAD compared to 10.55 ± 10.83 µm MAD for METHOD-B. The higher averages we gained for replenishing the solutions are seen in METHOD C, particularly where CS was used in the pre-treatment.

The pH value of freshly prepared PO_4_-Ringer’s solution was pH 7.05 ± 0.08 SD. The effect of adding the hardened cement paste pieces to the PO_4_-Ringer’s immediately increases the solution pH. At 24 h the solution pH values were recorded as pH 11.05 for CS, pH 9.26 for FL, and pH 11.38 for RG treated cement pastes after 24 h. However, when the Ringer’s solution was replaced after 24 h (keeping the same cement paste pieces) for CS, FL and RG, the pH values were 7.2, 7.07 and 9.97 respectively after a further 48 h (see Fig. S-3)). The lower pH in the replenished solution compared to the first solution is likely to be a result of the HAp coatings inhibiting further dissolution of the underlying cement paste phases.

### Thickest HAp layer from all treatment variables

The thickest HAp layer was recorded from PT_1_ (CS), S_1_ (0.5 Ringer’s) and METHOD A (24 h) at 41.92 µm ± 12.78 µm MAD. However, from this set of treatments we observed a greater degree of penetration of HAp into the cement paste matrix (i.e., a less distinct boundary between the HAp and the cement paste) and an incomplete surface coverage (Fig. 2, Table S3). Ideally, the protective layer should have an even surface coverage, and a relatively discrete interface with the hardened cement paste surface for subsequent removal during decommissioning. This was best produced by using colloidal silica with a replenished system, as in Method C. Since there was no obvious advantage in increasing solution concentration, thereafter experiments proceeded using solution S_1_. The combination of these treatments produced a relatively uniform and solid HAp layer with a thickness of 30.64 ± 21.38 µm MAD.

### HAp formation on the large cement paste block

**Figure 4:**
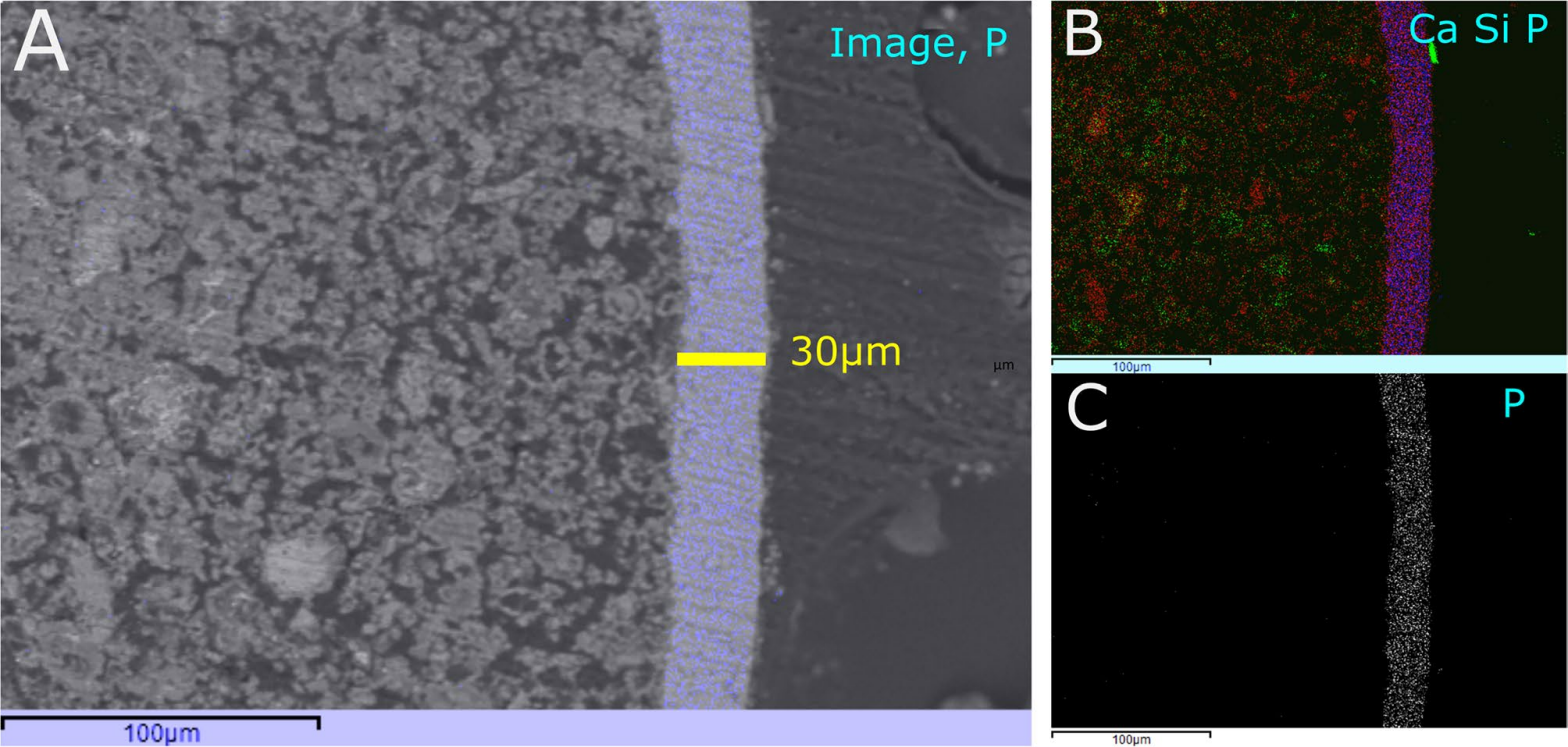
(**A**) SEM backscatter image of a cross-sectioned larger cement paste block showing HAp-layer in false-colour-blue. (**B**) composite RGB SEM–EDS element map of (**A**) showing Ca (red), Si (green), P (blue). (**C**) SEM–EDS Element map of P. Scale bars represent 100 μm . The average thickness of HAp layer in image (**A**) is 25.6 μm, and the average layer thickness of all four sides of the sectioned sample was determined as 20. 3 μm. See Fig. S-5 for other surfaces of this sample.

The resulting median thickness of the HAp layer shown in Fig. 4 on the larger hardened cement paste blocks (~ 40 × 47 × 48 mm) was 20.3 ± 8.4 µm MAD determined from three separate blocks (see Fig. 4, Fig S-4 to S-7, and Table S5). In all three samples, the HAp coating appeared to be evenly distributed with little or no large calcite or portlandite grains compared to earlier samples shown in Fig. 2. The notable difference between Figs. 2 and 4 in the distribution of calcite and portlandite at the cement's surface is likely due to removal during scrubbing with sand paper, prior to treatment, though the formation of secondary calcite during HAp formation is possible^42^.

### Estimating HAp thickness using solution chemistry

The (absolute) potential for HAp to form from a solution containing Ca and PO_4_ was calculated for thickness and compared to results gained experimentally (see SI-I Eq. (1)-(4)). The reported manufactured concentrations for the Ringer’s solutions were used for the calculations as they were found to be in good agreement with our measured concentrations by ICP-OES (see Table S6a-c). Table 1 shows a comparison of the calculated values for each ion to the experimental thickness. The data were derived from the three individual larger blocks subjected to treatments with solution S_1_ and METHOD-C, noting that in this experiment, all sides of the hardened cement paste were exposed due to the suspension of the block in the solutions.

**Table 1:** Calculated and measured experimental HAp thickness.

Block (cube) number*	Surface Area, A m^2^	Calculated HAp thickness using Eq. (3), Ca µm	Calculated HAp thickness using Eq. (4), P µm	Actual Experimental median HAp thickness µm	Measurement Deviations MAD
1	0.0118	2.66	18.25	14.5	± 2.66
2	0.0100	3.12	21.43	17.5	± 3.54
3	0.0103	3.05	20.92	24.2	± 3.70
Median 1–3				15.4	± 5.32

Conditions for the above were: Total concentration of solution S1 Used twice (Method–C). 0.24 g L^− 1^ CaCl_2_.6H_2_O. Ca = 0.087 g L^− 1^, *P *= 0.278 g L^− 1^.

*For block data and images see Figs. S-4 toS-7.

Applying the concentration of Ca supplied by the Ringer’s solution S_1_, to SI-I Eq. (3) underestimated the thickness of HAp, at 2.6 μm for large cement paste block 1 compared to the experimental median of 14.5 μm (*A* = 0.01178 m^2^). The difference in values implies that the known Ca in S_1_ was insufficient to provide the observed thickness, implying that source of Ca for HAp formation is via leaching from the hardened cement paste to achieve the thickness of HAp observed in our experiment. If, however, the concentration of P from S_1_ is applied to SI-I Eq. (4), the calculated thickness gives 18.25 μm, which is slightly larger than the mean observed HAp thickness of 14.5 μm. This calculated thickness of 18.25 μm implies that the PO_4_ in S_1_ was consumed and that Ca was not limiting, because it was supplied by the cement paste. Similarly, estimated thicknesses of 21.43 μm and 20.92 μm were calculated, compared to experimental thicknesses of 17.5 μm and 24.2 μm for cement paste blocks 2 and 3, respectively (see Table 1, Figs.S-4 to S-7). Therefore, the calculated thickness (based on the manufacturer’s specifications) provides a good approximation to the HAp thicknesses achieved experimentally for the larger cement paste blocks providing the counter ion (Ca or PO_4_) is not limiting. The HAp layers were thicker on the larger blocks and had good surface coverage (see e.g. Fig. S-8), compared to the smaller blocks in the first trial. This difference between the HAp layers might be explained by the larger blocks, with c. 10 × larger surface area, providing more Ca ions through leaching and roughening the surface prior to treatment.

### Strontium uptake on larger HAp coated cement paste cubes

**Figure 5:**
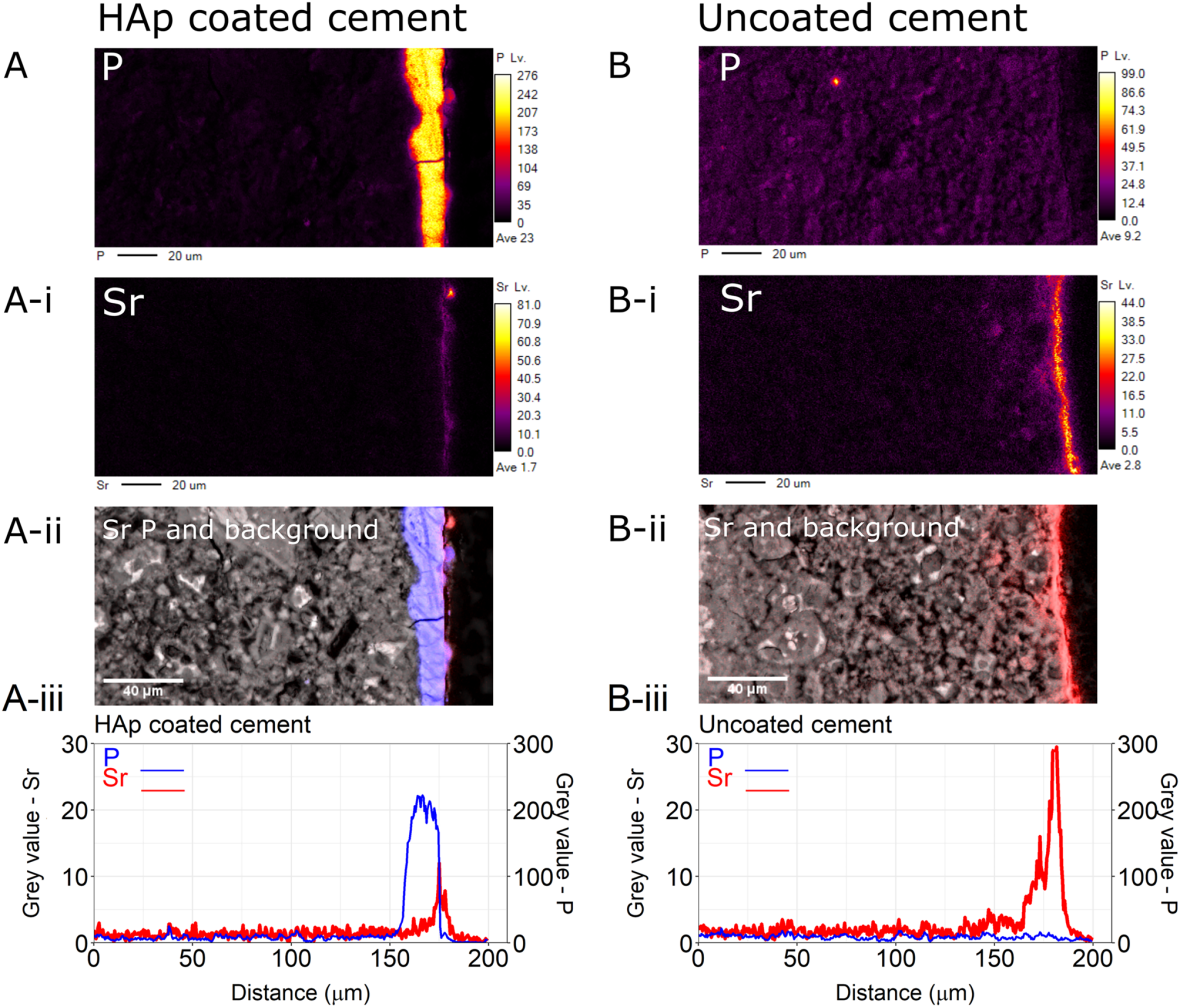
EPMA-WDX element maps showing; (**A**) cross section of the HAp coated cement paste after exposure to 1000 mg L^− 1^ Sr and (**B**) is the uncoated cement paste (control) after exposure to 1000 mg L^− 1^ Sr. The top images (**A**, **A**-**i**, **B** and **B-i**) are the element maps for P and Sr as labelled, scale bar is relative colour value intensity. (**A-ii**) and (**B-ii**) are the background image recorded from the backscatter detector image (greyscale) showing P (blue) and Sr (red) element maps overlaid. (**A-iii** and **B-iii**) is the whole image depth profile showing P (blue line) and Sr (red line). Scale bar for all images is 40 µm. HAp thickness determined from the width of the P band in the above image is 14.5 µm (see Table 1).

The layer of HAp on the larger cement paste blocks was determined to have a thickness of ~ 15 μm (see Table 1), made from a combination of ‘PT1, S_1_, METHOD-C’, as described above. These larger HAp-coated blocks were used for the determination of ingression of Sr into the cement paste. One HAp-coated block and an un-coated (control) cement paste block were each suspended in a 450 mL solution containing 1000 mg L^− 1^ Sr. One additional HAp-coated block was immersed in 450 mL containing 500 mg L^− 1^ Sr solution (Table S7a-c, Fig. S-9). After 192 h the concentration of Sr in the 1000 mg L^− 1^ solutions containing the coated and uncoated cement pastes had reduced to ~ 453 mg L^− 1^ and 564 mg L^− 1^ respectively—a reduction in Sr by 54.7% and 42.6%. Over the same period, for the HAp-cement paste block that had been in the 500 mg L^− 1^ Sr solution, the Sr in solution had reduced to 92.1 mg L^− 1^, demonstrating an 82% reduction in soluble Sr (Table S7a-c, Fig. S-9). The blocks were sectioned and examined for Sr ingress using EPMA-wavelength dispersive X-ray spectroscopy (EPMA-WDX) and the Sr Lα line using an accelerating voltage of 30 kV. Figure 5 shows cross-sectional images and depth profiles of the HAp-coated and uncoated cement paste blocks after exposure to 1000 mg L^− 1^ Sr. The image and depth profile of Sr in the uncoated cement paste (RHS of Fig. 5) shows that Sr has penetrated to a depth of at least 45 μm from the edge of the cement paste (Fig. 5). However, the Sr in the HAp-coated cement paste (LHS, Fig. 5), appears to be entirely contained within the boundary of the HAp layer; the graph in Fig. 5 shows Sr penetration into the HAp layer to a depth of about ~ 10 μm. The attachment of the HAp layer to the cement paste is perceived to be well-bonded, as evidenced by an image that shows a longitudinal crack in the HAp and not at the interface (see Fig. S-10); the crack was formed during sample preparation post-experiment.

## Discussion

Hydroxyapatite forms on pre-treated hardened cement paste blocks within hours of soaking in Ringer’s solution with added phosphate. Calcite is observed and its formation is suggested by solution equilibrium calculations (Geochemist’s Work Bench, *GWB,* Table S8a-e, Fig. S-11) as a secondary phase during HAp formation and also from the carbonation of portlandite (calcium hydroxide) in the hardened cement paste. Pre-treating the cement samples by soaking in a silica-based solution for 24 h significantly increases the rate of deposition and thickness of the HAp compared to using no silica. In our study, surface treatments of Colloidal Silica (CS) or Florisil suspensions resulted in thicker, more even, HAp layers compared to no silica pre-treatments, with CS showing the most potential. The CS suspension provides ligands with a negative surface charge for adhesion and nucleation of HAp to occur^33^. The importance of the ligands is supported by the weak formation of HAp on raw hardened cement pastes, although it also has a negatively charged surface. While ligands are likely to be imperative for nucleating HAp^31^, the nanoscale pore space between the ligands may also be important^43^. Hydrated CS, which is essentially SiO_2_ in equilibrium with Si(OH)_4_, has outwardly facing atoms in a tetrahedral shape which form silanol groups from the saturated hydroxyl ligands and condense into siloxane bridges (≡Si–O–Si≡)^44^. These silanol groups of the CS likely enabled better attachment and growth of HAp at the surface of the hardened cement pastes in this work. HAp was not observed to form on the smooth surfaces (i.e. surface formed directly in contact with the mould) of the cement pastes for any treatment. Roughening the surface of the pre-treated cement blocks had the effect of increasing the amount of aggregation of the colloidal silica suspension at the sample surface and therefore the amount of scaffold formed by silanol groups. The CS, which is normally stable in suspension, will aggregate on introduction of a counter-ion (usually added as a solution). In our experiment, the supply of the counterion (i.e., Ca^2+^) is liberated further from the cement as a result of the manual roughening of the surface. The increase in surface available Ca also likely facilitated HAp precipitation.

Both longer soaking times and solution replenishment increased HAp thickness, however increasing the strength of the Ringer’s solution whilst maintaining the same PO_4_ concentration was not advantageous. The saturation index for HAp is calculated at between 21 and 22 log Q/K at pH 10 (25 °C) for all our solutions (*GWB*, Table S8a-e, Fig. S11), which compares to between 7 and 8 log Q/K at pH 6, but the increase in ionic strength can affect the rate of reaction. If solution concentrations of Ca^2+^ are increased, then the reaction might proceed more quickly, in the bulk solution at the expense of surface mineralization. Lower concentrations of Ringer’s on the other hand give lower ionic strengths, permitting rapid leaching of Ca^2+^ ions from dissolution of hardened cement paste phases causing localized high concentrations of Ca^2+^ at or near the sample surface as well as increasing Ca^2+^ solution concentration^45–48^. Thus, maintaining a balance becomes important as sufficient free Ca^2+^ ions must be available to form a thick continuous HAp layer at the hardened cement pastes’ surface whilst keeping concentrations of Ca^2+^ in the bulk solution minimal to avoid HAp nucleation away from the surface. With respect to concretes, the presence of aggregates may affect the rate, thickness and coverage of HAp formation, due to a smaller surface area reducing the availability of leachable Ca^2+^ and would require further investigation. Layer thickness and porosity are important factors when considering barriers suitable against radionuclide ingression. While porosity was not determined experimentally, SEM images of the HAp layer of our proposed method displayed a dense coverage suggesting a low porosity. Current studies are investigating integrity of the HAp layer after drying and potential for additional precipitation of HAp to infill any surface imperfections e.g., microcracks and fissures.

A simple model predicting HAp thickness from solution concentrations using mineral density underestimated thickness using Ca concentrations that were in the Ringer’s solution. However, if the concentrations of P are accounted for and are applied to the model instead of Ca, (assuming Ca is not limited) there is better agreement to the median experimental HAp thicknesses. This implies that the theoretical Ca^2+^ concentration in the Ringer’s solutions alone are insufficient for HAp formation and that Ca^2+^ leaching from the cement substrate supplies sufficient Ca until P becomes limiting in solution with respect to HAp. The occurrence of Ca^2+^ leaching is confirmed by the rise in pH, likely due to portlandite dissolution from cement pastes. Subsequent HAp treatments applied to increase thickness, might require supplying more Ca^2+^ from solution, as the formation of a HAp layer will restrict Ca produced by cement phase dissolution. The agreement between the model using P and the experimental data indicates that the method proposed here is effective and efficient at producing HAp. The model can be used for scaling up to a larger scale operation and for tuning to a desired thickness, however both require further verification.

Exposing the HAp-coated cement to high concentrations of Sr indicates that HAp is a barrier to Sr when placed in solutions of 1000 mg L^− 1^ Sr. This Sr solution concentration used was above existing background concentrations in the cement samples (ppm concentrations are common in cement^6,49^). EPMA-WDX results in Fig. 5, show that the penetration of Sr is much less for the HAp-coated hardened cement pastes (~ 10 μm) than for the uncoated samples (~ 45 μm). Furthermore, the Sr in the HAp-coated samples was contained within the HAp layer and did not diffuse into the underlying cement substrate. Solution concentrations of Sr were reduced with the HAp-coated and the uncoated hardened cement pastes (Fig. S-9). The similar charge and ionic radii (1.2 Å and 1.0 Å, respectively) of Sr^2+^ and Ca^2+^ means that Sr^2+^ can be adsorbed onto HAp and that surface substitution with Ca^2+^ might subsequently occur^50^. In regular hydroxyapatite, Ca occupies two sites in the lattice which might be available for surface exchange with Sr^25^. In uncoated cement, the uptake of Sr^2+^ is controlled by C-S–H where from EXAFS analysis, Sr is bound by bridging O atoms to the surface sites of the C-S–H^6^. Competition for Sr therefore exists between the cement pastes and the HAp, while more research is required, our results imply that in HAp-coated cement pastes, HAp prevented the ingression of Sr into cement pastes from solution. The ability of HAp to adsorb and retain other radionuclides, anions or cations, requires further investigation though we might postulate that other divalent cations, might behave similarly to Sr^51^.

## Conclusion

In this work we show that HAp can be mineralised onto hardened cement pastes within a few days to a thickness of ~ 20.3 μm (range = 10–48 μm) from a phosphate buffered Ringer’s solution. The complex leaching chemistry of cement pastes means that careful balance of the P and Ca concentrations are needed to achieve maximum efficiency in HAp layer formation. We found that a solution of ½ strength Ringer’s amended with phosphate buffers (solution 1), which was replenished with the same solution after 24 h for a further two days, was the most successful treatment. HAp coverage was observed to be better on rough surfaces, with completely smooth surfaces showing no HAp growth for any treatment. That using a silica-based scaffolding technique was more effective than no surface treatment was, likely due to better HAp attachment via silanol-groups. Colloidal silica rather than Florisil was the preferred method for creating the scaffolding, based on performance (e.g. surface adhesion), availability and price. The average thickness of the HAp layers from the experiment roughly agreed with a density-based calculation using solution concentrations for a given surface area and showed that the treatment is not Ca limited, due to Ca leaching from hydrated cement phases. These calculations have potential to be used for scaling-up to larger surfaces and volumes. Preliminary testing of soluble Sr ingress showed that the HAp coating acts as a barrier, protecting the cement substrate from Sr contamination and confining the Sr to a thin surface layer within the HAp.

### Implementations

The ability to add layers of HAp rapidly and effectively onto hardened cement pastes surfaces has application for incorporating into future nuclear infrastructure as a design for minimising waste for decommissioning. HAp coating prevents radionuclides from entering the cement substrate, thus reducing future volumes of nuclear waste for subsequent treatment and disposal. Further investigation into HAp’s suitability is ongoing. For example, topics would include; increasing the thickness of the HAp; testing HAp formation and coverage on cements containing aggregates (e.g., concretes); investigating the long-term effect of radionuclides on the composition, structure and retaining properties of HAp; and developing a method for mechanical separation.

## Materials and Methods

### Preparation of the hardened cement pastes

Hardened cement paste coupons were prepared using (CEM-II/A-LL 32,5R), Hanson Heidelberg) and deionised water (DI) using a 2:5 wt./wt., liquid to solid, ratio and mechanically mixed for 15 min. According to the manufacturer’s chemical analysis, the product contains; SiO_2_(19.19%); Al_2_O_3_(4.57%), Fe_2_O_3_ (2.23%); CaO (66.5%); MgO (2.22%); SO_3 _(2.88%); K_2_O (0.67%), Na_2_O (0.21%); Cl (0.06%); Not detected (1.48%). The mixture was then poured into silicone moulds and left to set for 48 h under high humidity conditions before de-moulding. After curing at 100% relative humidity and ambient temperature (~ 20 °C) for 21 days, the coupons were rinsed with DI and rubbed by hand to remove surface material, then stored under ambient room conditions. Cement hydration was not arrested. No precautions were taken to prevent cement carbonation as this better describes the condition of cement that would be encountered during application of HAp to existing nuclear infrastructure. The small hardened cement-paste coupons used for the HAp coating experiments had an average coupon size of: L = 48 mm, B = 26 mm, H = 6 mm. Each of these small cement-paste coupons were subsequently sub-sectioned by slicing into four equal pieces using a diamond-tipped saw blade with resulting dimensions ~ 12 × 26 × 6 mm. All four sections were present at the start of each experiment and a section removed for analysis at each of the stated times.

The larger cement paste blocks (~ 40 × 47 × 48 mm) were prepared in the same way as described above with the following modifications. Nylon fishing line threaded with glass beads was embedded into the wet cement at one corner during curing to allow suspension of blocks in solution during treatment. After demoulding, the surfaces of the cement pastes blocks were roughened using P60 and P80 grade carbide-paper to encourage better adhesion of HAp. Blocks were constantly kept at 100% relative humidity (RH) until required and used within 1–2 months.

### Reagents and chemicals

MasterRoc® colloidal silica (CS) suspension part A (MP 320, BASF), was used without further modification at a concentration of 10 mL L^− 1^ (≡1.84 g L^− 1^ Si) of the product in pure water. Florisil® (FL), (MgSiO_3_, Fisons™), was used at a concentration of 10 g L^− 1^ (≡2.8 g L^− 1^ Si) in ultrapure water (Barnstead Nanopure, 18.2 MΩ cm^-3^). Florisil® [Magnesium silicate; CAS Number. 1343–88-0, MW = 100.39 g mol^− 1^] is a co-precipitated mixture of silica gel and magnesia comprising of ~ 84.0% SiO_2_, 15.5% MgO, and 0.5% Na_2_SO_4_ with a surface area of approximately 300 m^2^ g^− 1^ and is described as being largely insoluble.

Quarter strength Ringer’s solution (RG), (Oxoid) was prepared according to the manufacturer recommendations, which contains among other salts, CaCl_2_.6H_2_O at 0.12 g L^− 1^ i.e., 21.93 mg L^− 1^ (5.4^–4^ M) Ca (see Table S-6 for quantities). HAp forming solutions (S_1_, S_2_, S_3_) were formulated in multiples of 0.25 strength Ringer’s solution plus a phosphate buffer, such that the Ca solution concentrations were as follows; S_1_ = 0.5 Ringer’s, 0.0438 g L^− 1^ Ca; S_2_ = 0.75 Ringer’s, 0.065 g L^− 1^ Ca; S_3_ = Full-strength Ringer’s, 0.0877 g L^− 1^ Ca (Table S-6). Phosphate buffer (PO_4_) was comprised of a combination of monobasic (KH_2_PO_4_) and dibasic (K_2_HPO_4_) potassium phosphate salts used at 0.34 g L^− 1^ plus 0.348 g L^− 1^ respectively, with total P concentration of 0.139 g L^− 1^ (0.427 g L^− 1^ PO_4_). It was used at the same concentration in all S_1_, S_2_ and S_3_ solutions (Table S-6).

### HAp Batch Experiments

Reactions took place in lidded 100 mL glass (Duran-Schott) bottles without agitation. Figure 1 shows the schematic flow of the experiments. Blocks of three-week aged cement paste were first subjected to a two-day pre-treatment of one of; colloidal silica (CS), Florisil® (FL) (MgSiO_3_), or 0.25 Ringer’s solution (RG) as control for the scaffold formation (PT1, PT2, PT3). These blocks were then removed, sub-sectioned and placed in a phosphate plus Ringer’s solution (PO_4_-Ringer’s) (S1, S2, or S3). One block was retained for examination by SEM and XRD. The subsections were removed at 24 h (METHOD-A) and 72 h (METHOD-B) for examination of HAp formation (XRD, SEM). For METHOD-C sub-sectioned blocks were removed after 24 h and placed in an identical, fresh PO_4_-Ringer’s solution for a further 48 h. Conditions of the experiments are presented in Table S-1.

### HAp coatings on large hardened cement pastes blocks

For the ingression of Sr, the larger cement paste blocks were used. To make the HAp layer, the large cement paste cubes were first soaked in PT1, a 10 mL L^− 1^ CS suspension for 48 h, then transferred to solution S_1_ (0.5 strength Ringer’s plus phosphate buffer) for 24 h after which the solution was replenished and left for a further 48 h; i.e. ‘PT1 (Colloidal Silica), S_1_ and METHOD C’. To ensure exposure of all surfaces, cement pastes blocks were suspended in solutions using the embedded nylon (see above) line loosely covered with the lid and foil. Noting the observation that smooth surfaces inhibited HAp formation, the surfaces of the hardened cement pastes were roughened prior to pre-treatment exposing a fresh cement surface. This roughening increased the aggregation of the colloidal silica which was visible as a gel ~ 1 cm thick at the cement pastes surface in the pre-treatment phase (which was gently removed prior to the next treatment). Suspending the block, via a line embedded in the hardened cement pastes enabled exposure to all sides of the block during treatment. Following the HAp treatment, the HAp-coated blocks were maintained at 100% relative humidity before being suspended in Sr solution.

### Ingression of Sr into Hap-coated and uncoated cement pastes blocks

The HAp-coated cement paste blocks (described in the previous section) were suspended in 500 mL (*Nalgene*) plastic tubs containing solutions of 1000 ppm Sr as strontium chloride (SrCl_2_.6H_2_O) for 192 h. Aliquots (1 mL) of the solution were taken periodically, diluted, acidified and analysed using inductively coupled plasma-optical emission spectroscopy (ICP-OES).

### Analytical procedures

*Dry weights* and moisture contents of the cement pastes were calculated from triplicate measurements (4 pl. balance, Mettler Toledo) taken from cements dried at 40 °C and then 105 °C.

*Solutions* were diluted with 1% HNO_3_ and determined using ICP-OES (Thermo-Fisher). Solution pH was measured using a Mettler Toledo pH meter.

*Analysis of the solid samples* X-ray diffraction (XRD) patterns were collected from surfaces of whole cement pieces and on powdered bulk material using a Bruker D8 Advance X-Ray Diffractometer) (AMRL, University of Strathclyde). Cement pieces and finely ground powders were dried at 40 °C and stored in desiccators for at least 48 h before storage in sealed plastic containers prior to analysis. XRD patterns were collected using a Cu K-α anode (1.54 Å) at 40 kV, 40 mA with a Göbel mirror on the primary beam path, 1.0 mm divergence slit, ranging from 5 to 60, degrees 2Θ Cu K-α with a step size of 0.02 (2690 steps) and 0.5 s count time. Patterns were background subtracted, plotted using R and referenced to database entries from Rruff. The Rruff spectra used in this work are: aragonite R080142, calcite R04017 and hydroxyapatite R130713. TOPAS (v5.0, Bruker) was used for semi-quantitative Rietveld refinement using ICSD files (octa calcium phosphate: 27,050; calcite: 73,446; HAp: 187,840; aragonite: 32,100). Reflections from the sample holder were excluded from the refinement.

*Scanning electron microscopy-energy dispersive spectroscopy (SEM–EDS)* imaging, spectral analyses and element maps were performed on carbon-coated resin-embedded (Araldite2020) polished cross-sections using a Toshiba tungsten W-SEM equipped with Oxford-Inca EDS (AMRL, University of Strathclyde). For purposes of analysis in the SEM (and EPMA) chamber; the prepared specimens were dried at 40 °C for 24 h, then under vacuum at ambient temperature, and then stored in a desiccator until used for analysis. Individual element maps were collected as grey scale Tiffs at the same magnification (× 400 magnification). Samples exposed to 1000 mg L^− 1^ Sr was used due to lower detection limits with SEM (20 kV) and interference with Si at the Sr L line.

*Electron microprobe analysis, EPMA* on carbon-coated resin-embedded polished cross-sections used a JEOL, JXA-8530F Field emission EPMA equipped with 4 × wavelength-dispersive X-ray (WDX) spectrometers and energy-dispersive X-ray (EDS/ X) detector (University of Strathclyde). Element maps were acquired from tungsten wavelength dispersive X-ray (WDX) which were run simultaneous for Sr and P at 30 kV. Sr was acquired at both K and L-α lines respectively, however although the Bremsstrahlung background was present under the peaks obtained at lower energies, the Sr Lα line peaks were adopted for the elements maps as they were better defined than the Sr K peaks.

*Image processing was* performed using ImageJ on the collected SEM and EPMA element maps. HAp thicknesses were determined from the EDX phosphorus (P) maps (Table S-2) on calibrated images by drawing lines across the layer every 2–5 pixels and recording the length in imageJ. One to two mapped images (Table S-2) were acquired for each sample with between 67 and 373 measurements taken per image including where very thin or no layers existed, which were then averaged for statistical analysis. The profile line in Fig. 5 was produced by drawing a line of one pixel width halfway across the image, with the position recorded in a macro and using the plot profile tool (command: Analyze, Plot profile) to record the intensity of that line. This was repeated for all elements and then plotted together. To avoid compression loss the images were imported from the original excel files produced by the SEM or EMPA software and saved as 32-bit Tiffs.

*Composite images* and false red–green–blue (RGB) overlays were created by first converting the .Tiff images to a RGB colour then to RGB stack in ImageJ. All images, including the background image, were merged using the ‘merge’ tool. RGB channels were then dedicated to each element and finally exported as .png or .jpg format.

*Statistical analysis* of the P-layers was performed in R (base, plyr) with kurtosis and skewness calculated using MS-EXCEL. All plots and histograms were compiled and drawn in R (ggplot2). Data were tested for normality using the Shapiro and then medians and MAD are reported for the measurements.

## Supplementary Information

Supplementary Information 1.

Supplementary Information 2.

## Data Availability

All data generated or analysed during this study are included in this published article and its supplementary information files.
