# Supplementary material for: Hydroxyapatite coatings on cement paste as barriers against radiological contamination
Source: Sci Rep. 2023 Jul 10;13:11136. doi: 10.1038/s41598-023-37822-6 (PMC10333312; doi:10.1038/s41598-023-37822-6)
Supplement: Supplementary file 1 — Supplementary Information 1. [file 41598_2023_37822_MOESM1_ESM.docx]

Hydroxyapatite coatings on cement paste as barriers against radiological contamination

Supplementary Information: List of tables and figures

Contents

[Abstract 1](#_Toc127734505)

[Supplementary Information: List of tables and figures 2](#_Toc127734506)

**[Table S1](#_Toc127734507)** [Experimental Conditions 4](#_Toc127734507)

**[Fig. S-1](#_Toc127734508)** [XRD patterns for HAp layers 5](#_Toc127734508)

**[Fig. S-2](#_Toc127734509)** [XRD patterns for Method A and C 6](#_Toc127734509)

**[Table S2](#_Toc127734510)** [Quantitative results for XRD patterns:averages for each treatment type and summary table. 7](#_Toc127734510)

**[Table S3](#_Toc127734511)** [Images of SEM-EDS element maps for phosphorus (P) used for HAp depth measurements 8](#_Toc127734511)

**[Table S4](#_Toc127734512)** [Median HAp thickness measurements taken from P element maps in Table S3 11](#_Toc127734512)

**[Fig. S-3](#_Toc127734513)** [Solution pH during HAp formation. 12](#_Toc127734513)

**[Fig. S-4](#_Toc127734514)** [Schematic showing sectioning of cube1 shown in Fig.S-4 for SEM analysis. 13](#_Toc127734514)

**[Fig. S-5](#_Toc127734515)** [SEM backscatter images showing cross-section of cube1 with measurements. 14](#_Toc127734515)

**[Fig. S-6](#_Toc127734516)** [SEM backscatter images showing cross-section of cube2 with measurements. 15](#_Toc127734516)

**[Fig. S-7](#_Toc127734517)** [EPMA backscatter image showing cross-section of cube3 with measurements. 16](#_Toc127734517)

**[Table S5](#_Toc127734518)** [Combined HAp thicknesses for cement blocks and calculated thicknesses 16](#_Toc127734518)

**[Tables S6](#_Toc127734519)** [a-c solution compositions for Ringer’s Solutions 17](#_Toc127734519)

**[SI-I](#_Toc127734520)** [Calculations for thickness based on ion solution concentrations. 18](#_Toc127734520)

**[Fig S-8](#_Toc127734521)** [SEM image of surface of HAp on cement 19](#_Toc127734521)

**[Table S7](#_Toc127734522)** [a-c Solution chemistry during strontium exposure over 1 week 21](#_Toc127734522)

**[Fig. S-9](#_Toc127734523)** [Ion solution concentrations in during 1 week exposure of cement blocks to solutions of strontium chloride. 22](#_Toc127734523)

**[Fig. S-10](#_Toc127734524)** [EPMA-WDX , P element map showing a crack through the HAp. 23](#_Toc127734524)

**[Table S8](#_Toc127734525)** [a-d Equilibrium constants for Ringers solutions 24](#_Toc127734525)

**[Fig. S-11](#_Toc127734526)** [calculated solution data output plots(Geochemical Workbench) 27](#_Toc127734526)

## **Table S1** Experimental Conditions

| **Pre-Treatment** | **Solution type** | **Time in pre-treatment** | **HAp-treatment** | **Solution** | **Time in HAp-treatment** | **2nd Solution** | **Total HAp Time** |
| --- | --- | --- | --- | --- | --- | --- | --- |
| PT1 | CS | 2 days | Method A | S1 | 24 hrs | na | 24 |
|  |  |  | Method A | S2 | 24 hrs | na | 24 |
|  |  |  | Method A | S3 | 24 hrs | na | 24 |
| PT2 | FL | 2 days | Method A | S1 | 24 hrs | na | 24 |
|  |  |  | Method A | S2 | 24 hrs | na | 24 |
|  |  |  | Method A | S3 | 24 hrs | na | 24 |
| PT3 | RG | 2 days | Method A | S1 | 24 hrs | na | 24 |
|  |  |  | Method A | S2 | 24 hrs | na | 24 |
|  |  |  | Method A | S3 | 24 hrs | na | 24 |
|  |  |  |  |  |  |  |  |
| PT1 | CS | 2 days | METHOD-B | S1 | 72 hrs | na | 72 |
|  |  |  | METHOD-B | S2 | 72 hrs | na | 72 |
|  |  |  | METHOD-B | S3 | 72 hrs | na | 72 |
| PT2 | FL | 2 days | METHOD-B | S1 | 72 hrs | na | 72 |
|  |  |  | METHOD-B | S2 | 72 hrs | na | 72 |
|  |  |  | METHOD-B | S3 | 72 hrs | na | 72 |
| PT3 | RG | 2 days | METHOD-B | S1 | 72 hrs | na | 72 |
|  |  |  | METHOD-B | S2 | 72 hrs | na | 72 |
|  |  |  | METHOD-B | S3 | 72 hrs | na | 72 |
|  |  |  |  |  |  |  |  |
| PT1 | CS | 2 days | METHOD-C | S1 | 24 hrs | 48 hrs | 72 |
|  |  |  | METHOD-C | S2 | 24 hrs | 48 hrs | 72 |
|  |  |  | METHOD-C | S3 | 24 hrs | 48 hrs | 72 |
| PT2 | FL | 2 days | METHOD-C | S1 | 24 hrs | 48 hrs | 72 |
|  |  |  | METHOD-C | S2 | 24 hrs | 48 hrs | 72 |
|  |  |  | METHOD-C | S3 | 24 hrs | 48 hrs | 72 |
| PT3 | RG | 2 days | METHOD-C | S1 | 24 hrs | 48 hrs | 72 |
|  |  |  | METHOD-C | S2 | 24 hrs | 48 hrs | 72 |
|  |  |  | METHOD-C | S3 | 24 hrs | 48 hrs | 72 |
|  |  |  |  |  |  |  |  |

CS (Colloidal Silica); FL (Florisil); RG = (Ringer’s)

## **Fig. S-1** XRD patterns for HAp layers


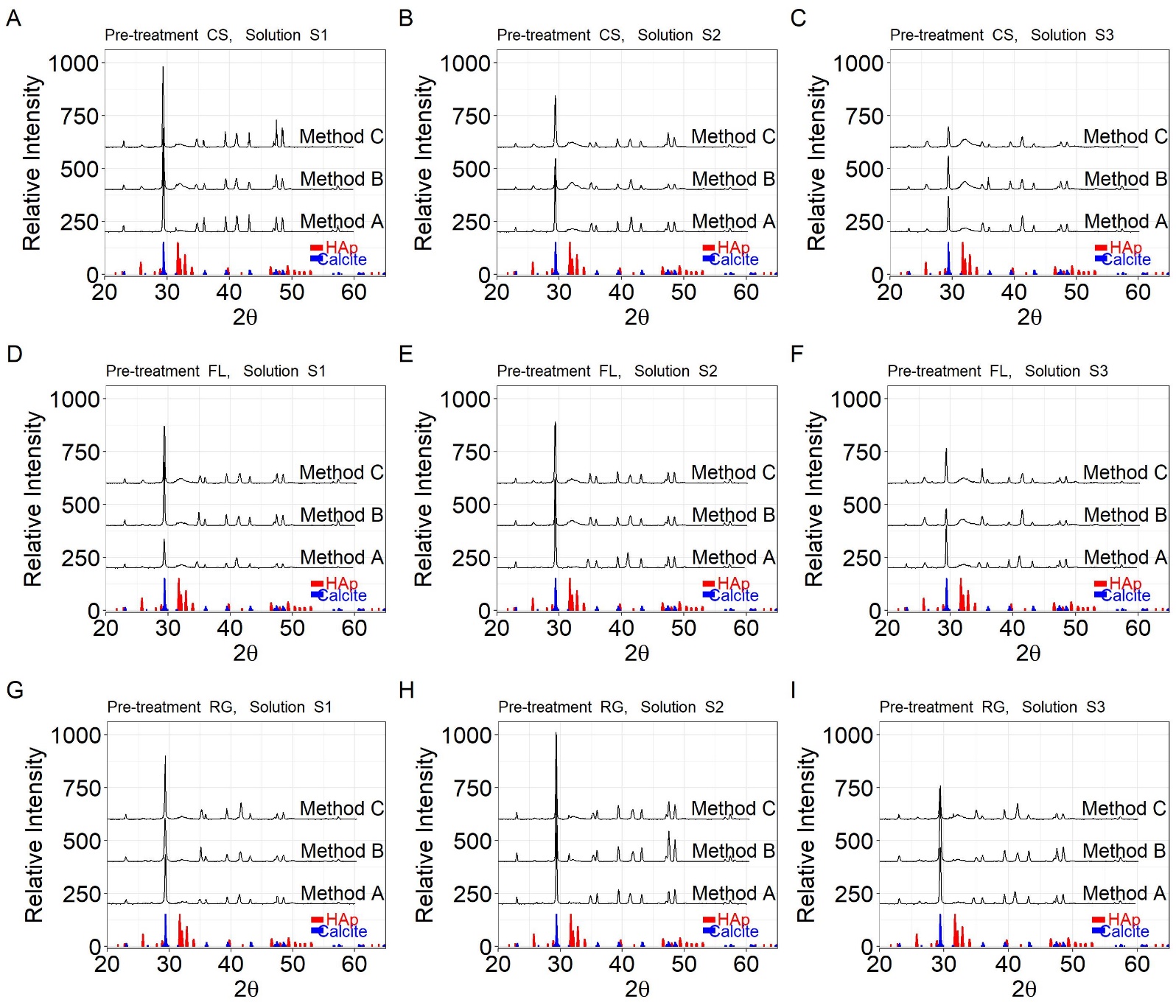


**Fig. S-1** X-ray diffraction (XRD) patterns taken from the surfaces of the cement after each treatments. Additional peaks were observed at 34.6-35.3 and 41.1-41.7 2Θ are an artefact from the Al sample holder and are labelled in Fig S-2.

## **Fig. S-2** XRD patterns for Method A and C


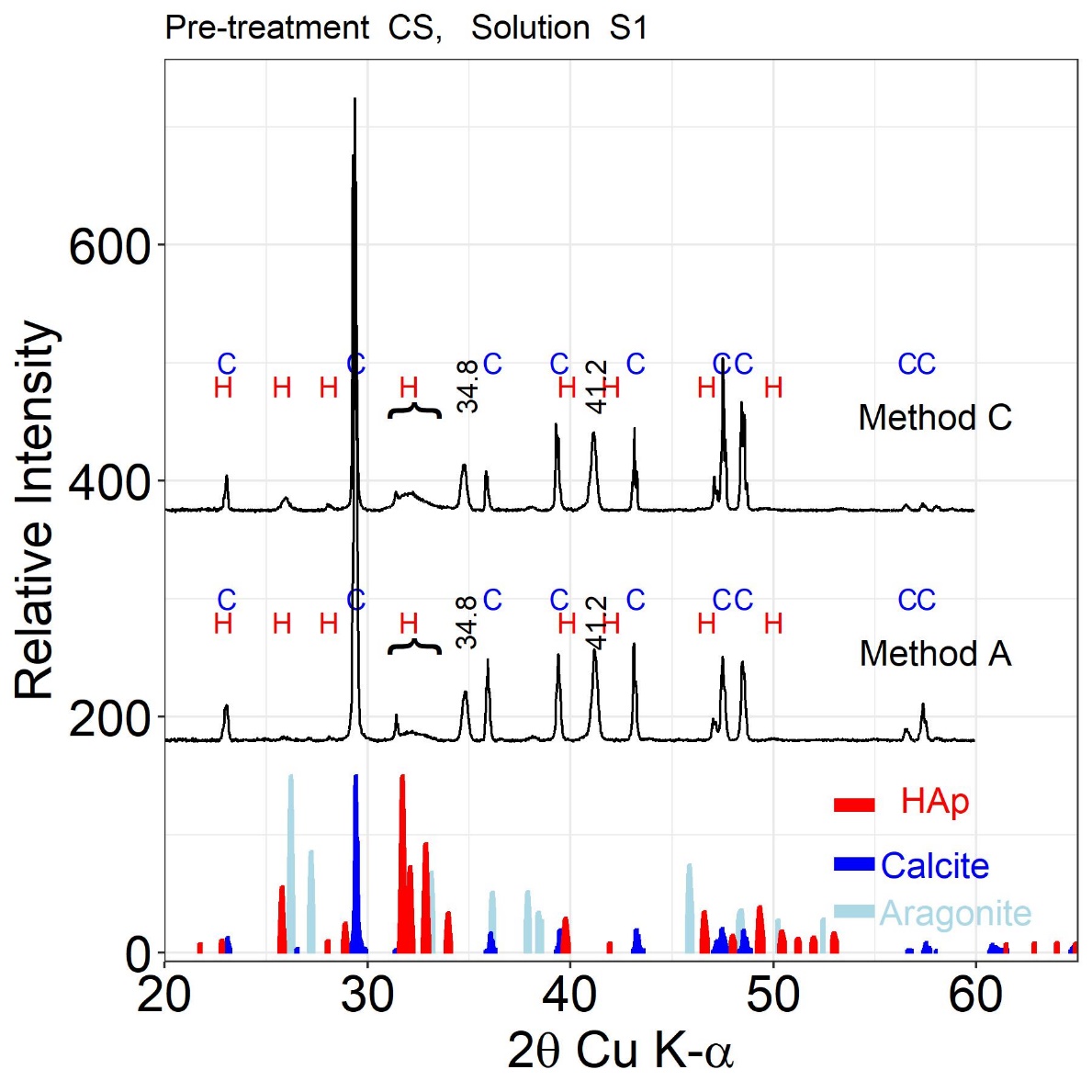


**Fig. S-2** XRD patterns of the HAp treated cement using PT1, Solution S1 (Solution 1), Method A compared to Method C. Additional diffraction peaks observed at 34.6-35.3 and 41.1-41.7 2Θ are an artefact from the Al sample holder and are therefore not considered as part of the sample. H represents HAp peaks, and C represents the calcite peak positions.

## **Table** **S2** Quantitative results for XRD patterns: averages for each treatment type and summary table.

| **Method** | **pre-treatment (PT)** | **solution** | **Calcite** | **HAp** | **Aragonite** | **OCP** | **Total** |
| --- | --- | --- | --- | --- | --- | --- | --- |
|  |  |  | **wt. %** | **wt. %** | **wt. %** | **wt. %** | **%** |
| METHOD A (24hrs) | PT1 (CS ) | S1 | 70.5 | 29.5 | <1 |  | **100.0** |
|  |  | S2 | 49.3 | 47.0 | 3.6 |  | **100.0** |
|  |  | S3 | 37.0 | 63.0 | n/a | * | **100.0** |
|  | PT2 (FL) | S1 | 33.3 | 66.7 | <1 |  | **100.0** |
|  |  | S2 | 66.4 | 29.5 | 4.14 |  | **100.0** |
|  |  | S3 | 38.5 | 54.2 | 7.31 | * | **100.0** |
|  | PT3 (RG) | S1 | 50.1 | 48.1 | 1.88 |  | **100.0** |
|  |  | S2 | 57.2 | 36.6 | 6.16 |  | **100.0** |
|  |  | S3 | 57.9 | 33.4 | 8.71 |  | **100.0** |
| METHOD B (72hrs) | PT1 (CS ) | S1 | 47.1 | 52.9 | n/a |  | **100.0** |
|  |  | S2 | 32.1 | 65.8 | 2.08 |  | **100.0** |
|  |  | S3 | 30.2 | 69.8 | n/a |  | **100.0** |
|  | PT2 (FL) | S1 | 52.7 | 46.2 | 1.05 |  | **100.0** |
|  |  | S2 | 54.5 | 43.6 | 1.86 |  | **100.0** |
|  |  | S3 | 30.4 | 63.7 | 5.91 |  | **100.0** |
|  | PT3 (RG) | S1 | 60.8 | 35.5 | 3.69 |  | **100.0** |
|  |  | S2 | 77.2 | 22.8 | n/a |  | **100.0** |
|  |  | S3 | 68.5 | 25.7 | 5.89 |  | **100.0** |
| METHOD C (24hrs + 48hrs) | PT1 (CS ) | S1 | 49.0 | 51.0 | n/a | ** | **100.0** |
|  |  | S2 | 44.7 | 55.3 | <1 |  | **100.0** |
|  |  | S3 | 21.3 | 78.7 | <1 |  | **100.0** |
|  | PT2 (FL) | S1 | 45.7 | 54.3 | n/a |  | **100.0** |
|  |  | S2 | 41.4 | 55.7 | 2.84 |  | **100.0** |
|  |  | S3 | 16.3 | 51.6 | 5.89 | 26.5 | **100.0** |
|  | PT3 (RG) | S1 | 48.4 | 51.6 | n/a |  | **100.0** |
|  |  | S2 | 61.7 | 36.8 | 1.54 |  | **100.0** |
|  |  | S3 | 39.1 | 59.6 | 1.31 |  | **100.0** |

Notes: CS = colloidal silica; FL = Florisil; RG = 0.25 % Ringer’s solution. S1 = PO_4_ + 0.5 Ringers, S2 = 0.75; Ringer’s; S3 = full Strength Ringer’s. calcite = CaCO3, HAp = hydroxyapatite Ca_6_(PO_4_)_10_.6H_2_O, Aragonite = (CaCO_3_), OCP = octa calcium phosphate Ca_8_H_2_(PO_4_)_6_ּ.5H_2_O. ICSD file numbers used for quantitative analysis: Octa calcium phosphate: 27050; Calcite: 73446; HAp: 187840; Aragonite: 32100. Aragonite is excluded at <1%

Table summary:

PT1(CS) and PT2(FL) have greater HAp that PT3(RG).

METHOD C had most HAp, (Method A = Method B)

Highest percentage of HAp was METHOD C, PT1, S3.

Also note high percentage of HAp in METHOD C, PT2, S3.

|  | **PT1** | **PT2** | **PT3** |
| --- | --- | --- | --- |
| **median** | 55.26 | 54.22 | 36.61 |
| **SD** | 14.54 | 10.72 | 12.19 |
| **mean** | 56.71 | 51.45 | 39.21 |
|  | **S1** | **S2** | **S3** |
| **median** | 50.68 | 43.59 | 59.55 |
| **SD** | 10.25 | 13.70 | 16.83 |
| **mean** | 48.08 | 43.72 | 55.57 |
|  | **METHOD A** | **METHOD B** | **METHOD C** |
| **median** | 47.03 | 46.37 | 54.36 |
| **SD** | 13.59 | 17.11 | 10.80 |
| **mean** | 45.12 | 47.17 | 55.09 |

## **Table S3** Images of SEM-EDS element maps for phosphorus (P) used for HAp depth measurements

| **METHOD-A – 24 hours** |  |  |
| --- | --- | --- |
| Sample ID | Image 1 | Image 2 (where taken) |
| PT 1 (CS)  Solution S1  (See Fig. 2) | 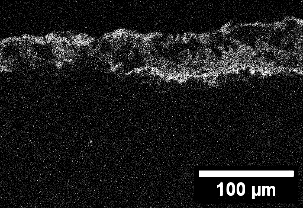 | 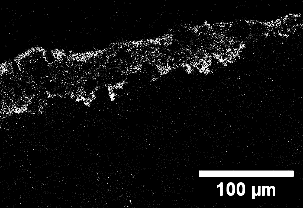 |
| PT 1 (CS)  Solution S2 | 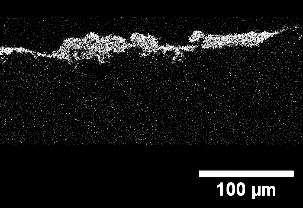 |  |
| PT 1 (CS)  Solution S3 | 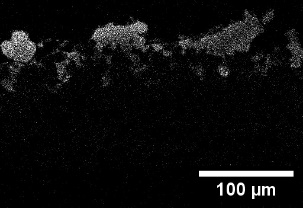 |  |
| PT 2 (FL)  Solution S1  (See Fig. 2) | 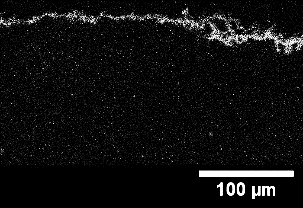 | 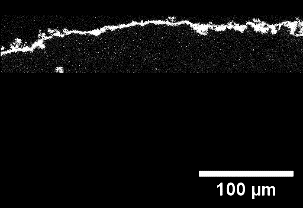 |
| PT 2 (FL)  Solution S2 | 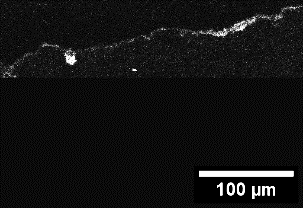 |  |
| PT 2 (FL)  Solution S3 | 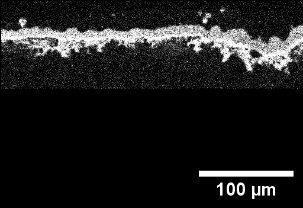 |  |
| PT 3 (RG)  Solution S1  (See Fig. 2) | 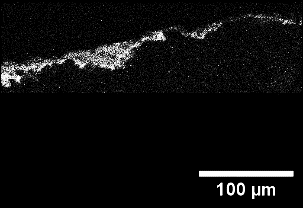 |  |
| PT 3 (RG)  Solution S2 | 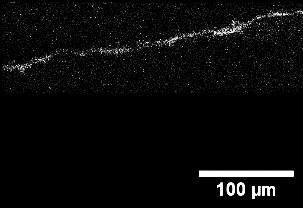 |  |
| PT 3 (RG)  Solution S3 | 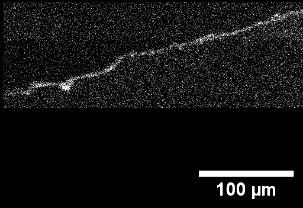 |  |

| METHOD-B 72 hours |  |  |
| --- | --- | --- |
| Sample ID | Image 1 | Image 2 (If taken) |
| PT 1 (CS)  Solution S1  (See Fig. 2) | 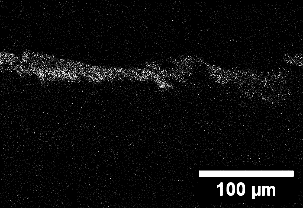 |  |
| PT 1 (CS)  Solution S2 | 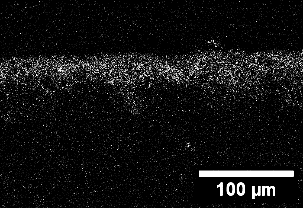 |  |
| PT 1 (CS)  Solution S3 | 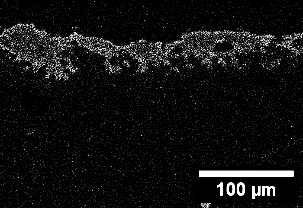 | 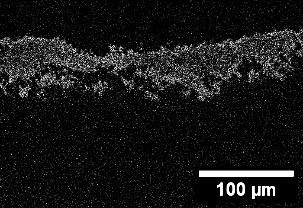 |
| PT 2 (FL)  Solution S1  (See Fig. 2) | 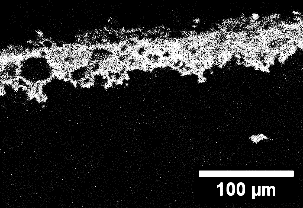 |  |
| PT 2 (FL)  Solution S2 | 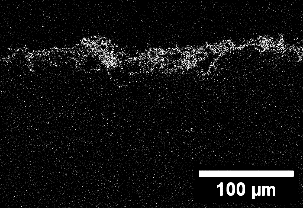 | 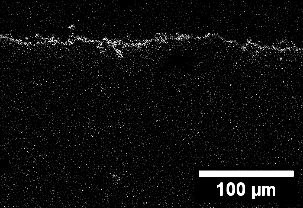 |
| PT 2 (FL)  Solution S3 | 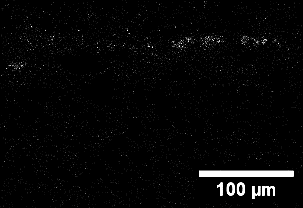 |  |
| PT 3 (RG)  Solution S1  (See Fig. 2) | 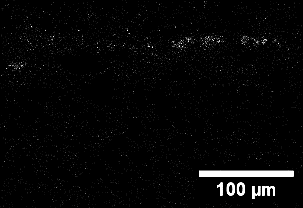 |  |
| PT 3 (RG)  Solution S2 | 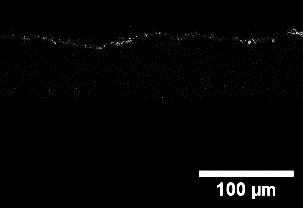 |  |
| PT 3 (RG)  Solution S3 | 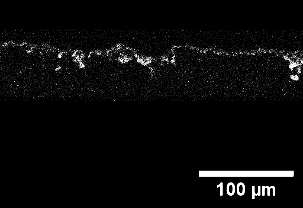 |  |

| METHOD-C – 72 hours |  |  |
| --- | --- | --- |
| Sample ID | Image 1 | Image 2 (If taken) |
| PT 1 (CS)  Solution S1  (See Fig. 2) | 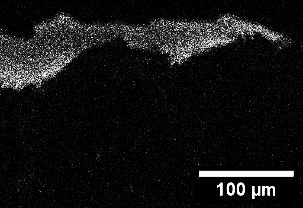 |  |
| PT 1 (CS)  Solution S2 | 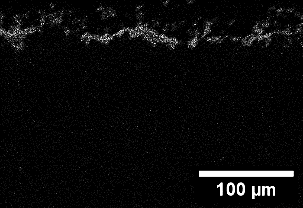 |  |
| PT 1 (CS)  Solution S3 | 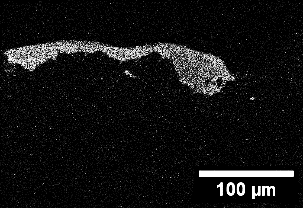 | 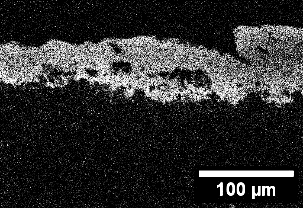 |
| PT 2 (FL)  Solution S1  (See Fig. 2) | 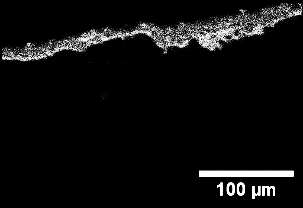 |  |
| PT 2 (FL)  Solution S2 | 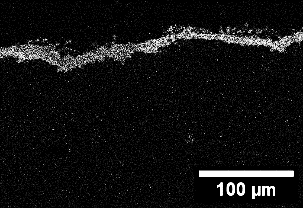 | 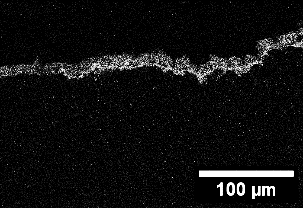 |
| PT 2 (FL)  Solution S3 | 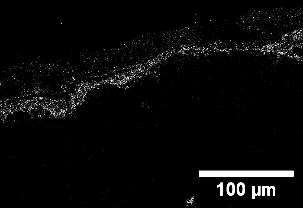 |  |
| PT 3 (RG)  Solution S1  (See Fig. 2) | 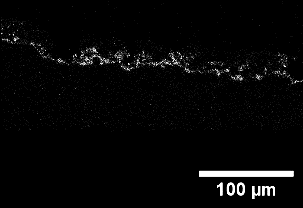 |  |
| PT 3 (RG)  Solution S2 | 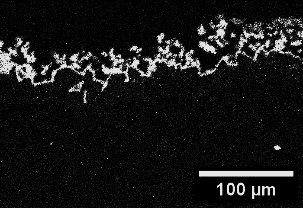 |  |
| PT 3 (RG)  Solution S3 | 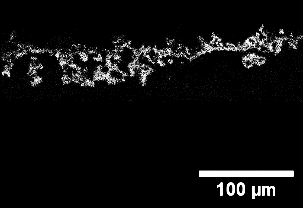 |  |

## **Table S4** Median HAp thickness measurements taken from P element maps in Table S3

| **Rank** | **Pre-treatment** | **HAp solution** | **HAp Method** | **Duration** | **Median** | **MAD** | **Min** | **Max** | **n** |
| --- | --- | --- | --- | --- | --- | --- | --- | --- | --- |
|  |  |  |  |  | **μm** | **μm** | **μm** | **μm** |  |
| 1 | CS | S1 | A | 24hr | 41.92 | 12.78 | 5.31 | 70.32 | 202 |
| 2 | FL | S1 | B | 72hr | 39.03 | 8.92 | 23.96 | 58.31 | 109 |
| 3 | CS | S3 | C | 72hr | 35.57 | 28.29 | 3.47 | 82.69 | 73 |
| 4 | CS | S1 | C | 72hr | 30.64 | 21.83 | 2.1 | 63.9 | 112 |
| 5 | CS | S2 | B | 72hr | 27.18 | 6.04 | 15.91 | 44.81 | 89 |
| 6 | CS | S3 | B | 72hr | 26.17 | 14.04 | 5.52 | 57.82 | 67 |
| 7 | CS | S1 | B | 72hr | 17.36 | 5.58 | 4.63 | 32.39 | 78 |
| 8 | RG | S3 | C | 72hr | 15.93 | 12 | 3.23 | 44.73 | 98 |
| 9 | FL | S1 | C | 72hr | 15.32 | 6.01 | 4.35 | 29.21 | 105 |
| 10 | CS | S2 | C | 72hr | 15.04 | 11.65 | 1.45 | 40.07 | 191 |
| 11 | FL | S3 | A | 24hr | 14.02 | 4.55 | 6.65 | 35.85 | 107 |
| 12 | CS | S3 | A | 24hr | 13.44 | 15.9 | 0.58 | 53.78 | 73 |
| 13 | FL | S2 | C | 72hr | 12.72 | 3.86 | 3.93 | 25.75 | 141 |
| 14 | FL | S3 | C | 72hr | 11.43 | 6.01 | 5.2 | 31.81 | 110 |
| 15 | CS | S2 | A | 24hr | 11.25 | 8.11 | 0.99 | 27.81 | 373 |
| 16 | FL | S2 | B | 72hr | 9.27 | 6.03 | 0.87 | 32.75 | 48 |
| 17 | RG | S1 | C | 72hr | 9.25 | 6.59 | 0.29 | 23.14 | 43 |
| 18 | RG | S2 | C | 72hr | 7.9 | 6.13 | 1.56 | 33.25 | 116 |
| 19 | RG | S1 | A | 24hr | 6.15 | 4.74 | 1.83 | 26.89 | 58 |
| 20 | FL | S1 | A | 24hr | 5.62 | 3.69 | 0.99 | 34.44 | 97 |
| 21 | FL | S3 | B | 72hr | 5.23 | 3.49 | 0.58 | 13.61 | 98 |
| 22 | RG | S2 | A | 24hr | 5.2 | 2.55 | 1.16 | 13.55 | 103 |
| 23 | RG | S3 | B | 72hr | 4.89 | 2.22 | 0.52 | 19.07 | 86 |
| 24 | FL | S2 | A | 24hr | 4.49 | 2 | 0.42 | 15.45 | 97 |
| 25 | RG | S2 | B | 72hr | 4.29 | 3.23 | 0.26 | 12.69 | 55 |
| 26 | RG | S3 | A | 24hr | 4.14 | 1.59 | 1.76 | 11.94 | 85 |
| 27 | RG | S1 | B | 72hr | 3.88 | 1.52 | 0.26 | 9.82 | 120 |

MAD = Median absolution deviation

## **Fig. S-3** Solution pH during HAp formation.


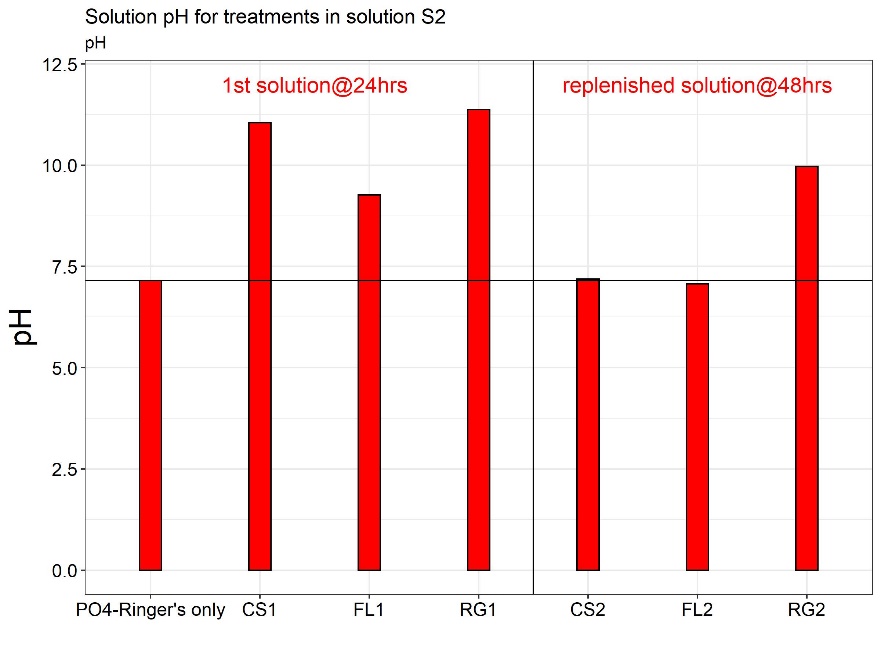


Fig.S-3 shows pH values of the PO_4_-Ringer’s solutions at initial pH and with the cement pieces; after 24 hours (C1, FL1 and RG1): and in the replenished solution after 48 hours (CS2, FL2 and RG2). Where the PO_4_-Ringer’s was replenished with a fresh solution after 24 hours. The horizontal bar references the pH of the starting solutions. Data points are single measurements taken after instrument stabilisation time.

Geochemical modelling predicts that HAp will form between pH 6- and 12 and calcite forms over pH 8 (see Fig. S-8). At pH >10, calcite formation is less favoured, but HAp will continue to form. The starting pH of the colloidal silica was higher than the FL, favouring HAp formation. However, both pH values were lower after soaking in the second PO_4_-Ringer’s solution, possibly because HAp coating was already preventing portlandite dissolution, which was contributing to the higher pH.

## **Fig. S-4** Schematic showing sectioning of cube1 shown in Fig.S-4 for SEM analysis.


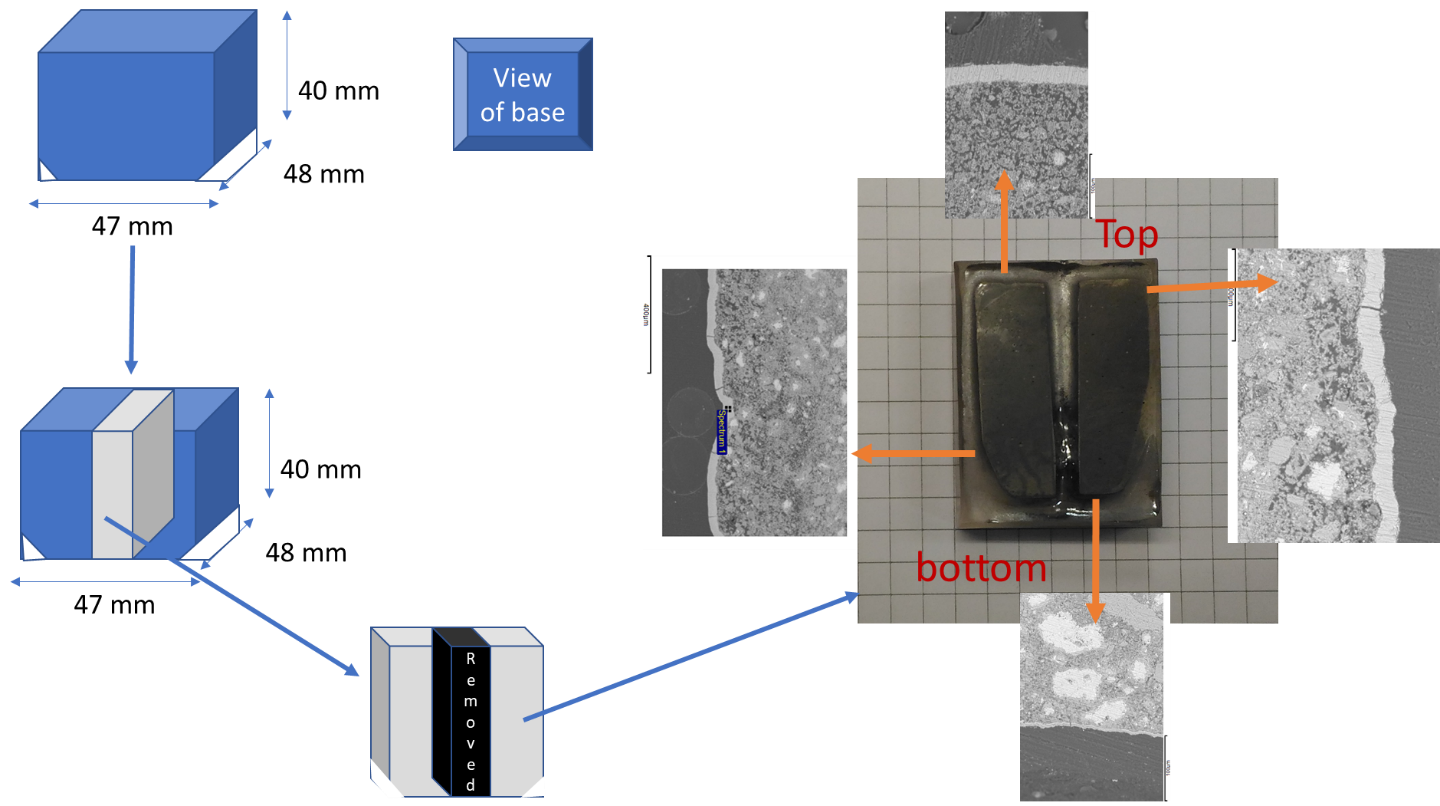


Fig. S-4 Schematic showing sectioning of cube1 shown in Fig.S-5for SEM analysis. The ‘bottom’ and ‘top’ of the cube represent how the cube was positioned in the reaction solutions during Hap formation. Thus gravity may have affected the thickness of the HAp at various locations.

## **Fig. S-5** SEM backscatter images showing cross-section of cube1 with measurements.


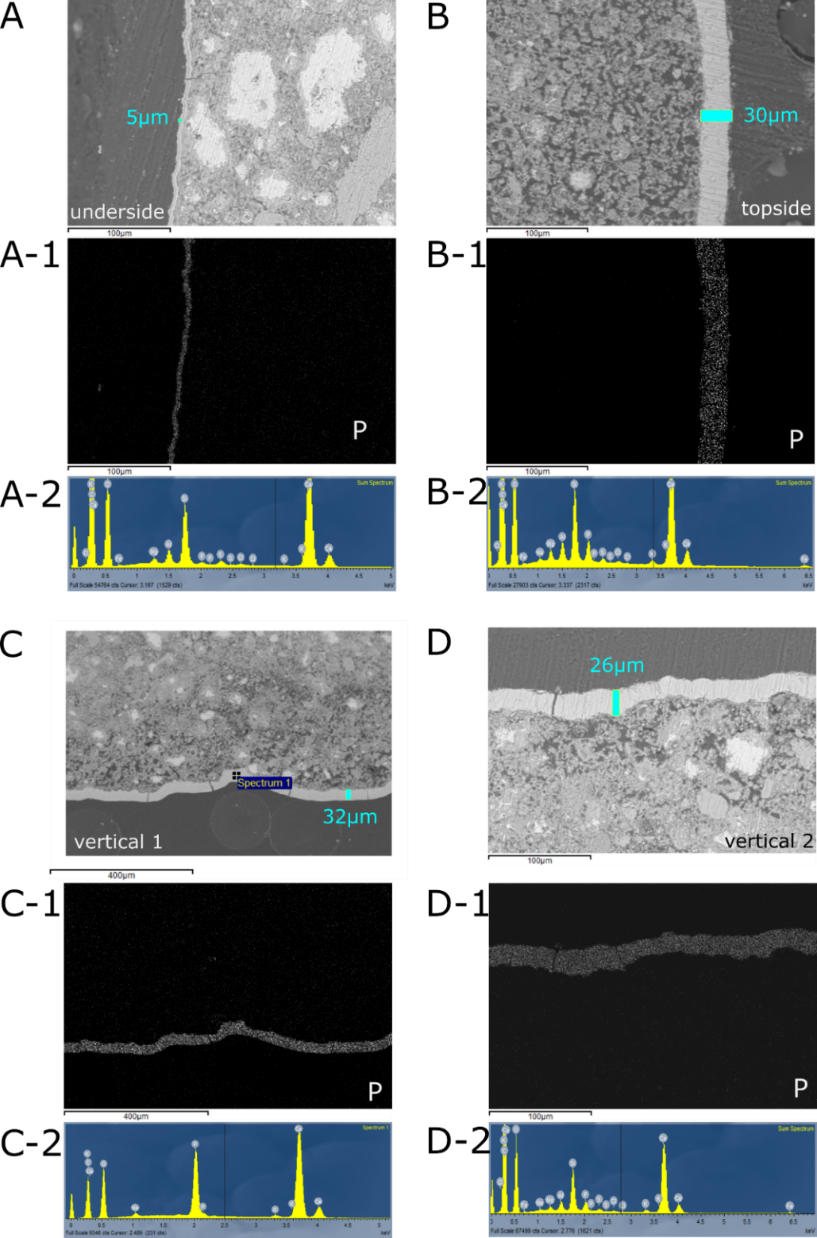


| Image / cube | Median | MAD | Mean | SD | Min | Max | Number of measurements, n |
| --- | --- | --- | --- | --- | --- | --- | --- |
| A-1 | 5.0 | 2.7 | 5.1 | 1.2 | 2.3 | 8.8 | 48 |
| B-1 | 25.6 | 1.3 | 25.5 | 1.7 | 20.6 | 30.0 | 70 |
| C-1-vertical | 28.5 | 2.5 | 28.6 | 3.7 | 19.9 | 38.9 | 89 |
| D-1 | 20.4 | 0.4 | 20.7 | 3.1 | 10.7 | 26.9 | 86 |
| combined | 24.2 | 2.66 | 21.7 | 8.4 | 2.3 | 38.9 | 293 |

(E) HAp thickness values A-D corresponding to Fig. S-5 (above)

Figure S-5 Scanning electron microscope (SEM) image and element P map and energy dispersive EDS (Energy Dispersive Spectroscopy) spectra, for the larger (pre-roughened) cement blocks. Note that image orientation is not representative of the cubes’ position in the reaction container. (A-C) are SEM images (A-1 to D-1) are the EDS elements maps for P (A-2 to D-2) are the EDS sum spectra (A-2, B-2, D-2) or a point spectrum (C-2) taken from the P-rich area. All scale bars represent 100 μm except for image C which is 400 microns. Data are average of ‘n’ measurements taken across the P-rich area using ImageJ. (E): table of measurement values for cube 1. Figure S-4 is the schematic demonstrating sample preparation.

## **Fig. S-6** SEM backscatter images showing cross-section of cube 2 with measurements.


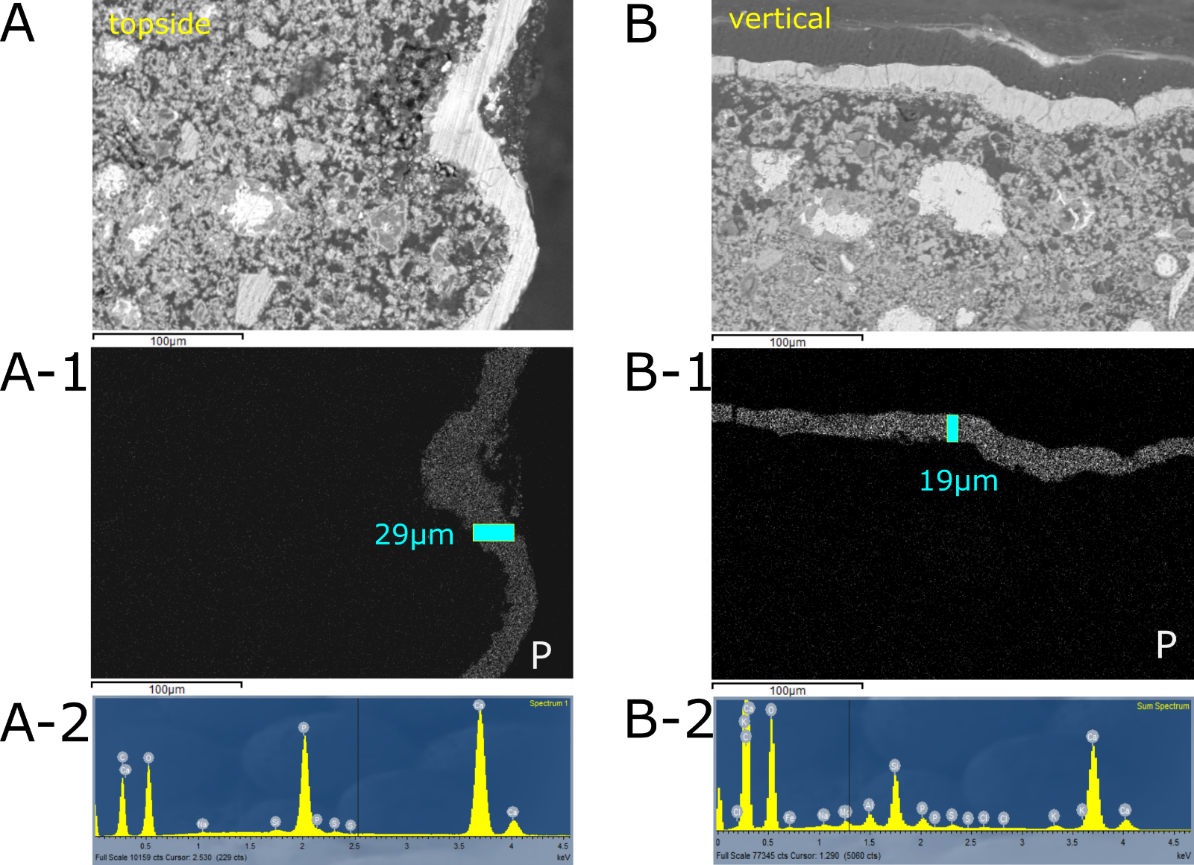


| Image / cube | Median | MAD | Mean | SD | Min | Max | Number of measurements, n |
| --- | --- | --- | --- | --- | --- | --- | --- |
| A-1-Topside | 25.7 | 6.1 | 27.4 | 8.6 | 16.1 | 48.8 | 67 |
| B-1-vertical | 15.7 | 2.1 | 15.56 | 3.58 | 1.4 | 25.44 | 112 |
| Combined | 17.5 | 3.5 | 20.0 | 8.4 | 1.4 | 48.8 | 179 |

Cube 2 HAp thickness values from images A, B

Fig. S-6 caption: SEM image and element P map and energy dispersive spectra SEM-EDS, for the larger (pre-roughened) cement blocks. Note that image orientation is not representative of the cubes’ position in the reaction container. (A, B) are SEM images; (A-1, B-1) are the EDS element maps for P; (A-2) EDS sum point spectrum from the P-rich area, (B-2) EDS sum spectrum of whole image. Scale bars represent 100 μm; (C ) Data are average of ‘n’ measurements taken across the P-rich area using ImageJ.

## **Fig. S-7** EPMA backscatter image showing cross-section of cube3 with measurements.

(A)
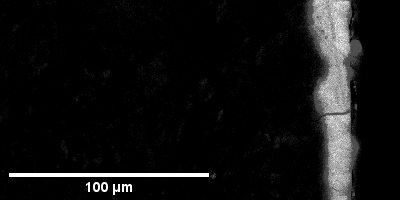


| Image / cube | Median | MAD | Mean | SD | Min | Max | Number of measurements, n |
| --- | --- | --- | --- | --- | --- | --- | --- |
| HAp-coated cube in Sr experiment | 14.5 | 14.5 | 15.4 | 3.2 | 10.8 | 20.7 | 57 |

(B) Table of cube 3 HAp thickness measurement data from image A

Fig. S-7(A) EPMA WDX (wavelength dispersive X ray)- image of P layer in the HAp-coated large cement cube that was used for the strontium soaking experiment which was sanded before HAp application. (B)HAp thickness measurements taken from (A).

## **Table S5** Combined HAp thicknesses for cement blocks and calculated thicknesses

|  | **-1-Cement block**  **Figure S-5** | **-2-Cement block**  **Figure S-6** | **-3-HAp thickness in cement block Figure S-7**  **(Sr experiment)** | **Combined data**  **Figures S5, S6, S7** |
| --- | --- | --- | --- | --- |
| **Median** | 24.2 | 17.5 | 14.5 | ***20.3** |
| **Min** | 2.3 | 1.4 | 10.8 | 1.4 |
| **Max** | 38.9 | 48.8 | 20.7 | 48.8 |
| **n** | 293 | 179 | 57 | 529 |
|  |  |  |  |  |
| **Cube dimensions** |  |  |  |  |
| **LBH** | 47.06×46.7×31.38 | 47.3×46.9×29.72 | 48.26×47.3×37.35 |  |
| **Surface Area,**  **A cm^2^** | 102.8 (0.0102 m^2)^ | 100.35 (0.010m^2)^ | 117.8 (0.011703 m^2)^ |  |
| **Calculated P –depth from model** (using P) in microns** | 20.91 | 21.43 | 18.37 |  |

*The median thickness of the HAp layer shown in Fig. 4 on the larger cement blocks was **20.3** ± 8.4µm MAD

** see additional information on the calculating the thickness of Hap from input solutions at the end of this document

## **Tables S6** a-c solution compositions for Ringer’s Solutions

### Table S6a Manufacturers ingredients ¼ strength Ringers in 1 litre solution

| Salt | Mass of salt in ¼ strength (g per litre) |
| --- | --- |
| NaCl | 2.25 |
| NaHCO_3_ | 0.05 |
| KCl | 0.105 |
| CaCl_2_.6H_2_O | 0.12 |

### Table S6b calculated elemental concentrations for each solution S1, S2 or S3

| **Element** | **¼ strength Ringer’s**  **mg L ^-1^** | **S1**  **2 × ¼**  **e.g. solution 1**  **mg L ^-1^** | **S2**  **3×¼**  **e.g. solution 2**  **mg L ^-1^** | **S3**  **4 × ¼**  **e.g. solution 3**  **mg L ^-1^** |
| --- | --- | --- | --- | --- |
| Ca | 21.93 | 43.93 | 65.78 | 87.85 |
| Cl | 1458.9 | 2907.34 | 4361.01 | 5814.67 |
| K | 308.96 | 364.18 | 419.24 | 474.31 |
| HCO_3_ | 36.32 | 72.63 | 108.95 | 145.26 |
| Na | 898.8 | 1797.6 | 2696.4 | 3595.2 |
| Phosphate additions to solutions | | | | |
| P | - | 139.13 | 139.13 | 139.13 |
| PO_4_ | - | 427.00 | 427.17 | 427.17 |

Ca is calculated as CaCl_2_.6H_2_0; P and PO_4_ are calculated from K_2_HPO_4_ and KH_2_PO_4_ at 0.3482 and 0.34 mg L^-1^ respectively

### Table S6c Analysis of phosphate amended Ringer’s solutions by ICP-OES

|  |  | **Ca** | **K** | **P** | **pH** |
| --- | --- | --- | --- | --- | --- |
|  | Solution strength | **mg L^-1^** | **mg L^-1^** | **mg L^-1^** |  |
| **Solution, S1** | x2 ringers | 41.66 | 411.2 | 145.62 | 7.15 |
| **Solution, S2** | x3 ringers | 56.58 | 482.6 | 143.38 | 7.01 |
| **Solution, S3** | x4 ringers | 79.98 | 569 | 138.62 | 7.0 |

## **SI-I** Calculations for thickness based on ion solution concentrations.

Using the dimensions of the surface that the HAp is to be deposited on coupled with the solution chemistry of the proposed treatment, it is possible to calculate the potential thickness of the HAp layer as described below.

For example, without taking thermodynamics or dissolution into account and if all solutes form minerals i.e with respect to HAp, the potential thickness is calculated using mineral density of HAp onto a given surface area from a known solution chemistry as follows (Eq.1):

*b = V / A (1)*

where *b* = thickness (metres), *V* = Volume of solid HAp produced, m^3^, and *A* is the surface area, m^2^, of the cement on which HAp is deposited. The volume, *V*, is derived from the theoretical mass (*m*, kg) and density (*ρ, kg m^-3^*) of hydroxyapatite (Eq.2.):

*V* = *m* / *ρ (2)*

The density of a resulting mineral, *ρ*, in equation (2) is used to predict thickness, where the density of HAp, is 3160 kg m^-3^ (3.16 g cm^-3^) with the total mass of HAp (kg) being calculated from its molecular mass, MM, 502.31 g mol^-1^. To work out the contribution of Ca or P to the resulting thickness in any given reagent the fraction of ‘Ca’ or ‘P’ from the empirical formula [Ca_5_(PO_4_)_3_(OH)] is applied as follows.

*m[HAp]* = *m*[Ca] / (5 (MM[Ca]) / MM[HAp] ) (3)

*m[HAp]* = *m*[P] / (3 (MM[P]) / MM[HAp] ) (4)

In equation (3), the HAp thickness is calculated from the mass of available Ca in solution, i.e. from CaCl_2_.6H_2_O dissolved (measured or theoretical) in the Ringer’s solution (L) of the reagent, e.g. ([CaCl_2_.6H_2_O] × Vol) × (MM[Ca] / MM [CaCl_2_.6H_2_O]. Alternatively, using Eq. (4) HAp thickness can be calculated according to the amount of known P in solution. P is calculated from the added amount of phosphate buffer added as mono and di-basic potassium phosphate, assuming Ca is in excess.

## **Fig S-8** SEM image of surface of HAp on cement


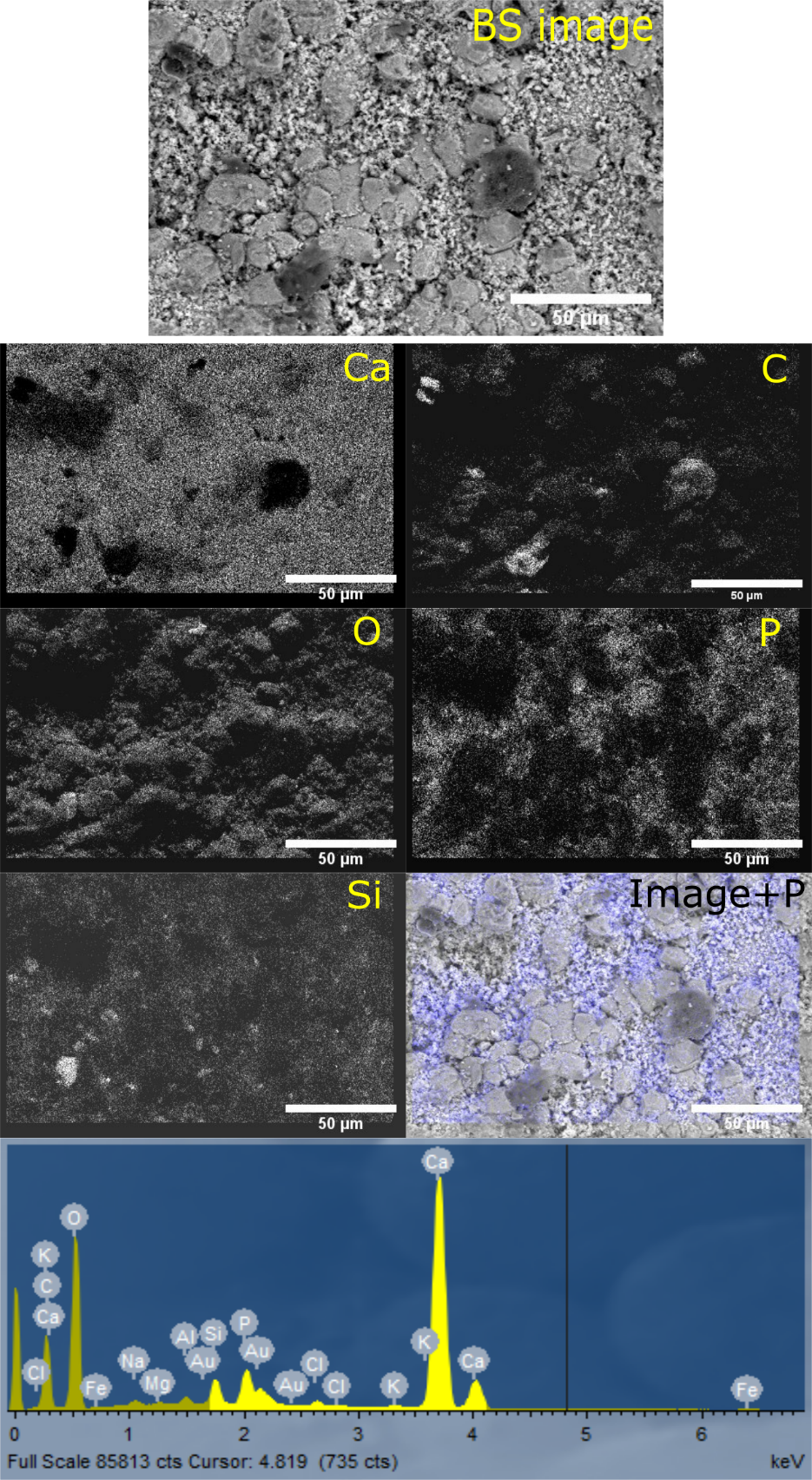


Figure caption; SEM image of surface of cube; right hand side shows the distribution of P coloured blue , which represents the hydroxyapatite layer .

## **Table S7 a-c** Solution chemistry during strontium exposure over 1 week

Starting solution is 1000 mg L^-^^1^ or 500 mg L^-1^ made from SrCl_2_.6H_2_O. First sampling was at 10 minutes, T = 0.6. Solutions measured using ICP-OES.

**Table S7a** Strontium solution concentration for HAp-coated cement in starting solution of 1000 mg L^-1^ Sr

| **Time** | **HAp-coated Ca** | **HAp-Coated P** | **HAp Coated Sr** |
| --- | --- | --- | --- |
| **hrs** | mg L^-1^ | mg L^-1^ | mg L^-1^ |
| 0.6 | 2.934 | 0.165 | 1016.0 |
| 48 | 102.4 | 0.131 | 855.40 |
| 120 | 134.4 | 0.063 | 727.20 |
| 192 | 145.4 | 0.063 | 453.00 |

**Table S7b** Strontium solution concentration for uncoated cement in a starting solution of 1000 mg L^-1^ Sr

| **Time** | **Uncoated Ca** | **Uncoated P** | **Uncoated Sr** |
| --- | --- | --- | --- |
| **hrs** | mg L^-1^ | mg L^-1^ | mg L^-1^ |
| 0.6 | 4.661 | 0.063 | 985.4 |
| 48 | 235.8 | 0.063 | 833.2 |
| 120 | 282.7 | 0.602 | 736.1 |
| 192 | 290.3 | 0.11 | 564.6 |

**Table S7c** Strontium solution concentration for the HAp-coated cement in starting solution of 500 mg L^-1^ Sr

| **Time** | **HAp coated -500 Ca** | **HAp coated -500 P** | **HAp coated -500 Sr** |
| --- | --- | --- | --- |
| **hrs** | mg L^-1^ | mg L^-1^ | mg L^-1^ |
| 0.6 | 2.03 | 0.175 | 512.5 |
| 48 | 55.05 | 0.063 | 313.4 |
| 120 | 59.38 | 0.063 | 186.5 |
| 192 | 68.61 | 0.063 | 92.1 |

## **Fig. S-9** Ion solution concentrations in during 1 week exposure of cement blocks to solutions of strontium chloride.

See Table S 7 for data.


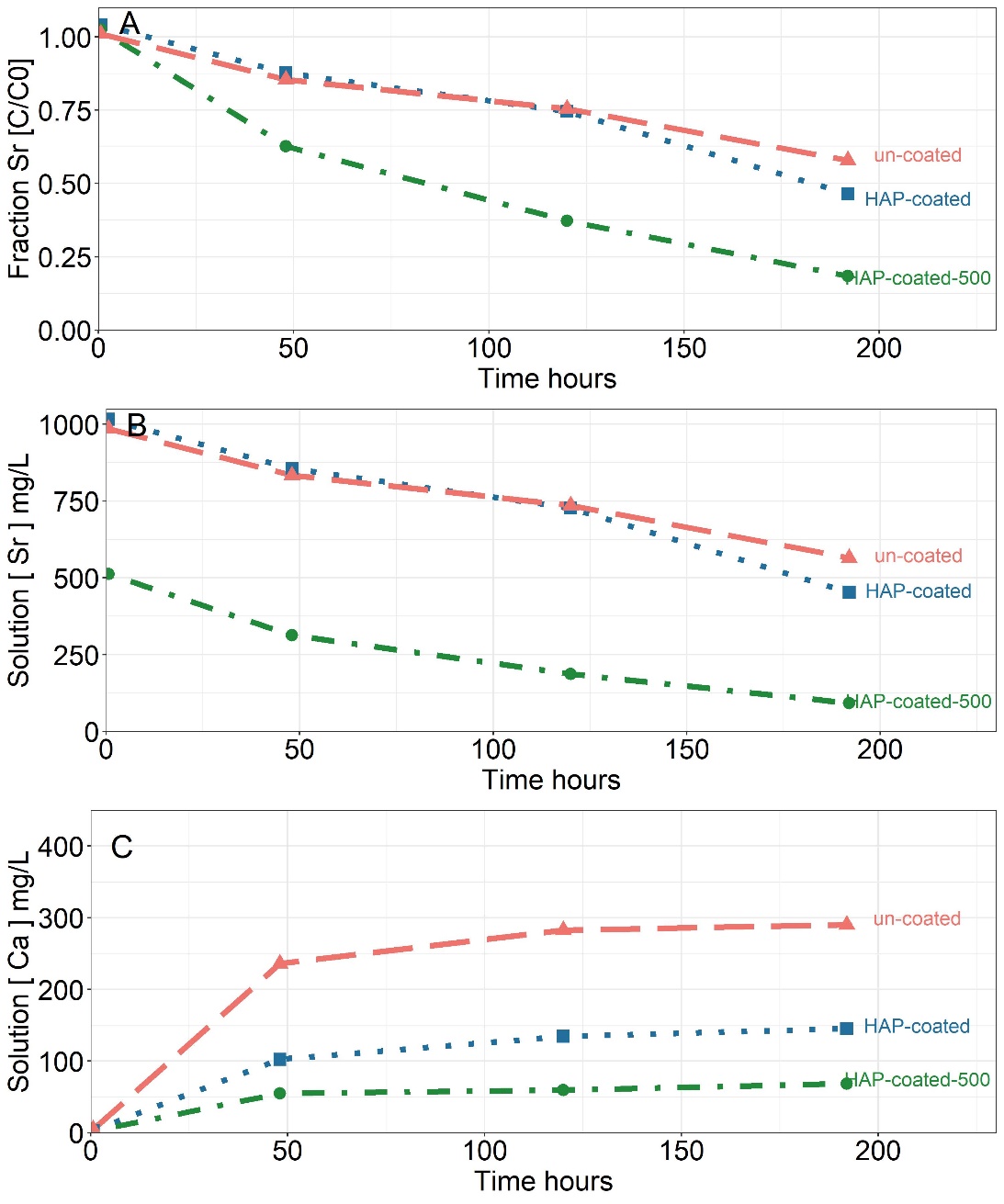


Fig.S-9 Loss of Sr from solution in the presence of uncoated (control) cement (red, dashed) in a 1000 mg L^-1^, HAp-coated cement (blue, dots) in a 1000 mg L^-1^ and HAp-coated cement (green, dash-dot-dash) in a 500 mg L^-1^ Sr solution. Where (A) fraction loss of Sr, (B) same data set as in (A) but shown in mg L^-1^ (C) solution concentration for calcium.

## **Fig. S-10** EPMA-WDX , P element map showing a crack through the HAp.

The crack mostly likely occurred during sample preparation or in the vaccum chamber of the EMPA.


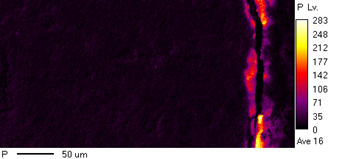


Fig S-10 EPMA-WDX image above showing the element map of P demonsrates that the HAp is sequrely anchored to the cement since the crack, which we belive occurred during sample preparation (e.g. cutting, drying, resin embedding, drying and viewing under vaccum), appears though the middle of the HAp region.

## **Table S8** a-d Equilibrium constants for Ringer’s solutions

**Table S8-a** Input concentrations used for GWB modelling.

|  | Ca  mg L-^1^ | Cl  mg L^-1^ | K  mg L^-1^ | Na  mg L^-1^ | PO_4_  mg L^-1^  (as HPO_4_) | HCO_3_ | pH values  (for each value between) |
| --- | --- | --- | --- | --- | --- | --- | --- |
| Sol1 | 43.86 | 2907.34 | 364.18 | 1797.6 | 427.17 | 72.63 | 6 – 12* |
| Sol2 | 65.89 | 4361.01 | 419.24 | 2696.4 | 427.17 | 72.63 | 6 – 12 |
| Sol3 | 87.85 | 5814.67 | 474.31 | 3595.2 | 427.17 | 72.63 | 6 – 12 |
| Sol1 +Ca | 87.85 | 2985.04 | 364.18 | 1797.6 | 427.17 | 72.62 | 6 – 12 |

- Note; this represent the range of pH calculated for in the model i.e pH 6, 7, 8, 9, 10, 11 and 12

### Tables S8 b-d Geochemists Workbench (GWB) Output

| Table S8b Solution S1 (Ringer’s 0.5 strength) | | | | |
| --- | --- | --- | --- | --- |
| pH | Ionic strength | Hydroxyapatite | Calcite | Aragonite |
|  | IS (molal) | SI (log_Q/K) | SI (log_Q/K) | SI (log_Q/K) |
| 6 | 0.090 | 7.201 | -2.624 | -2.789 |
| 7 | 0.092 | 12.763 | -1.307 | -1.472 |
| 8 | 0.093 | 16.989 | -0.295 | -0.460 |
| 9 | 0.093 | 20.132 | 0.506 | 0.341 |
| 10 | 0.092 | 21.096 | 0.675 | 0.510 |
| 11 | 0.094 | 20.604 | 0.083 | -0.082 |
| 12 | 0.105 | 20.288 | -0.558 | -0.723 |

| Table S8c Solution S2 (Ringer’s 0.75 strength) | | | | |
| --- | --- | --- | --- | --- |
| pH | Ionic strength | Hydroxyapatite | Calcite | Aragonite |
|  | IS (molal) | SI (log_Q/K) | SI (log_Q/K) | SI (log_Q/K) |
| 6 | 0.132 | 7.682 | -2.344 | -2.508 |
| 7 | 0.134 | 13.181 | -1.025 | -1.190 |
| 8 | 0.135 | 17.405 | -0.003 | -0.168 |
| 9 | 0.134 | 20.693 | 0.829 | 0.664 |
| 10 | 0.134 | 22.003 | 1.067 | 0.902 |
| 11 | 0.136 | 21.724 | 0.521 | 0.356 |
| 12 | 0.146 | 21.470 | -0.097 | -0.262 |

| Table S8d Solution S3 (Ringer’s 1 strength) | | | | |
| --- | --- | --- | --- | --- |
| pH | Ionic strength | Hydroxyapatite | Calcite | Aragonite |
|  | IS (molal) | SI (log_Q/K) | SI (log_Q/K) | SI (log_Q/K) |
| 6 | 0.174 | 7.990 | -2.151 | -2.315 |
| 7 | 0.176 | 13.427 | -0.833 | -0.998 |
| 8 | 0.176 | 17.641 | 0.194 | 0.029 |
| 9 | 0.176 | 21.022 | 1.044 | 0.879 |
| 10 | 0.175 | 22.597 | 1.338 | 1.173 |
| 11 | 0.177 | 22.559 | 0.856 | 0.691 |
| 12 | 0.187 | 22.384 | 0.267 | 0.102 |

| Table S8e Solution S1 plus CaCl_2_.6H_2_O | | | | |
| --- | --- | --- | --- | --- |
| pH | Ionic strength | Hydroxyapatite | Calcite | Aragonite |
|  | IS (molal) | SI (log_Q/K) | SI (log_Q/K) | SI (log_Q/K) |
| 6 | 0.093 | 8.673 | -2.328 | -2.4928 |
| 7 | 0.095 | 14.216 | -1.010 | -1.1745 |
| 8 | 0.095 | 18.433 | 0.005 | -0.1596 |
| 9 | 0.094 | 21.588 | 0.821 | 0.6565 |
| 10 | 0.093 | 22.732 | 1.062 | 0.8966 |
| 11 | 0.094 | 22.407 | 0.536 | 0.3707 |
| 12 | 0.103 | 22.119 | -0.093 | -0.2583 |

## **Fig. S-11** calculated solution data output plots(Geochemical Workbench)


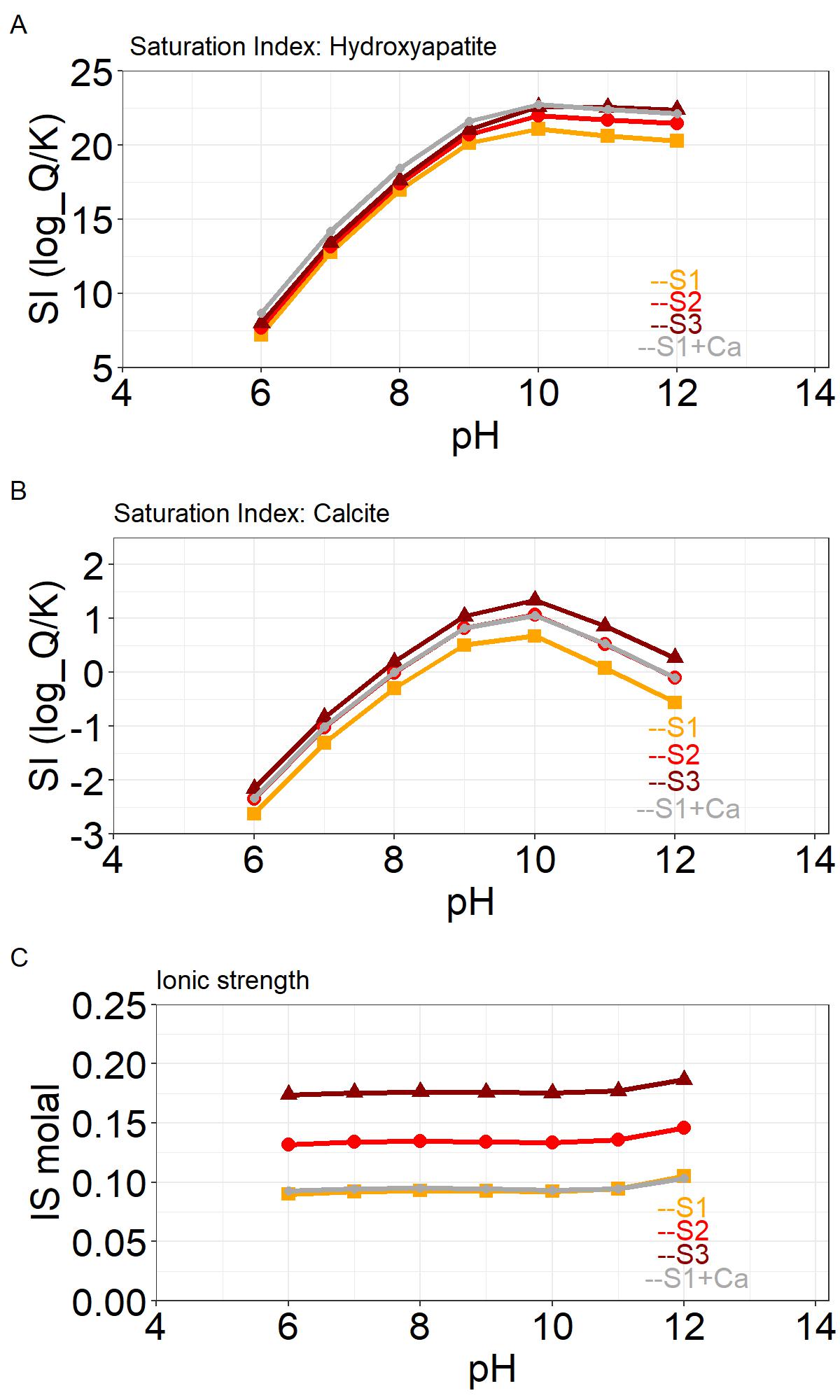


Fig.S-11. Output plots for Geochemists’ Workbench (GWB) for the PO_4_-Ringer’s solutions (S1, S2, S3) and solution S1 with calcium chloride added to the same concentration as S3, as a function of pH. Data represent the solution only without substrate. A) shows the saturation indices for hydroxyapatite; B) shows the saturation indices for calcite C) solution Ionic strength (molal). For corresponding data see Table S8 a-e.
